# Estimating and Evaluating Roughness Length and Displacement Height in Heterogeneous Urban Environments

**DOI:** 10.1007/s10546-026-00968-7

**Published:** 2026-04-06

**Authors:** Jason P. Horne, Ying Pan, Kenneth J. Davis

**Affiliations:** Department of Meteorology and Atmospheric Science, University Park, PA USA

**Keywords:** Aerodynamic roughness length, Displacement height, Anemometric methods, Morphometric methods, Urban micrometeorology

## Abstract

The roughness length ($z_0$) and displacement height ($z_d$) are essential surface-layer parameters in numerical models (e.g., weather, climate, wall-modeled LES, etc.). This work evaluates the consistency of $z_0$ and $z_d$ estimates from morphometric and anemometric methods using data from two eddy-covariance flux towers (AmeriFlux US-INg and US-INc) in Indianapolis, IN. Results show inconsistencies in estimated $z_0$ and $z_d$ values depending on the chosen method. The two evaluated anemometric methods estimate non-physical values of $z_d$ when compared to roughness elements surrounding both towers. Additionally, predictions of mean wind speed using surface-layer similarity theory with morphometric estimates exhibit a bias during near-neutral and stable conditions relative to observations. The overestimation of mean wind speed by surface layer similarity theory is consistent with previous observational and modeling studies in urban areas, suggesting that the application of similarity theories to urban environments may have limitations. Differentiation of vegetation from built structures appears to impact morphometric $z_0$ and $z_d$ estimates, particularly where vegetation is abundant; however, it has little impact on correcting biases in the similarity theory. Specifically, we find that existing similarity theories using morphometric estimates underestimate integral velocity and length scales, and the degree of underestimation depends on the stability conditions. Accounting for the degree of anisotropy in surface-layer turbulence helps reduce the biases between similarity theories and observations during unstable conditions, but not in near-neutral cases. Future work is needed to identify the cause of such biases for near-neutral conditions.

## Introduction

In weather or climate models, the parameterization of the surface-layer stress-strain-rate relationships, typically represented via some form of surface layer similarity theory, usually employs two aerodynamic parameters: the displacement height ($z_d$) and the roughness length ($z_0$). These variables affect the characteristic length scales of turbulent exchanges of momentum, heat, and mass between the surface and the atmosphere (Garratt 1992). The displacement height can be considered an adjusted “ground surface” “felt” by the mean flow (Oke et al. 2017; Monin and Obukhov 1954; Garratt 1992), or the height of average drag force exerted by roughness elements (REs) (Jackson 1981; Thom 1971). The roughness length, $z_{0}$, is related to the characteristic size of REs (Jackson 1981; Zannetti 1990; Wieringa 1986).

In the urban environment, three major approaches have been developed to estimate $z_{d}$ and $z_{0}$ which are reviewed by Grimmond and Oke (1999): i) reference-based methods, which use published look-up tables or figures, ii) anemometric methods, which use anemometer measurements and similarity theories and iii) morphometric methods, which use the morphology of built structures and vegetation. Previous studies have used in-situ observations and either anemometric or morphometric methods to estimate $z_0$ and $z_d$ in urban areas [e.g., Liu and Sun (2010): Nanjing, China, Al-Jiboori and Fei (2005): Beijing, China, Rooney (2001): Birmingham, UK]

Among various anemometric methods (see Kent et al. (2017a), Table 5), the two most commonly applied methods for urban areas are the temperature variance method TVM; first introduced by , (Rotach, 1994) and wind variance method WVM; first introduced by , (Toda and Sugita, 2003). Both TVM and WVM start from estimating $z_{d}$ using similarity scaling for unstable conditions. The TVM method uses the temperature variance scaling, while the WVM method uses the vertical velocity variance scaling. Toda and Sugita (2003) reported that both methods were helpful for estimating $z_d$ using data collected by a single sonic anemometer. With changing wind directions, the WVM method was found more sensitive to changes in $z_d$ compared to the TVM method. After obtaining $z_d$, both TVM and WVM estimate $z_0$ by matching observations for near-neutral conditions with the logarithmic mean-velocity profile (Stull 1988; Grimmond et al. 1998). This practice assumes that $z_d$ does not vary with stability, which might not be realistic (Zilitinkevich et al. 2008).

Morphometric methods are largely built upon the foundation of Macdonald et al. (1998), who developed a model based on the expression derived by Lettau (1969) and experimental data of flows over staggered cuboid arrays. This model was expanded by Kanda et al. (2013) to incorporate large-eddy simulation (LES) results of flows over the urban districts of Tokyo and Nagoya, Japan. Recently, Kent et al. (2017b) incorporated the impacts of vegetation in the Macdonald et al. (1998) and Kanda et al. (2013) formulation. Kent et al. (2018b) found that when vegetation cover is included in the formulation, $z_0$ increased and $z_d$ decreased in the winter compared to the summer, particularly in vegetated urban areas like suburban neighborhoods or parks.

Previous studies show discrepancies between morphometric and anemometric estimates of $z_0$ and $z_d$ in urban areas. A comparison between multiple morphometric and anemometric estimates was performed by Kent et al. (2018b) at two vegetated suburban sites (Swindon, UK, and Seoul, South Korea). Kent et al. (2018b) show that estimates of both $z_0$ and $z_d$ obtained using anemometric methods were consistently larger than those obtained using morphometric methods. Furthermore, they show that of the morphometric methods the Kanda et al. (2013) estimates were consitently greater and aligned better with anemometric estimates. da Silveira et al. (2022) compared the anemometric and morphometric methods in São Paulo, finding, similar to Kent et al. (2018b), that the morphometric methods of Kanda et al. (2013) estimated $z_d$ values twice as large as other morphometric methods. da Silveira et al. (2022) also note when including the methods of Kent et al. (2017b) where vegetation impacts are considered, Kanda et al. (2013) $z_d$ estimates show the best agreement with anemometric methods.

Given the diversity of urban environments within and across cities, the associated estimates of aerodynamic parameters need continued investigation and comparison to previous findings. Furthermore, these aerodynamic parameters should be evaluated within surface layer similarity theory and compared with in situ measurements, as they are most relevant to the atmospheric science community in this context. This work assesses the differences in $z_{0}$ and $z_{d}$ estimates obtained using various anemometric and morphometric methods and investigates how these estimates may indicate limitations in applying similarity theories to urban environments. We obtain estimates of $z_{0}$ and $z_{d}$ using multiple anemometric and morphometric methods at a suburban site and a downtown site in Indianapolis, IN (observational data and analysis procedures are explained in Section 2), compare our results to those reported for other cities [e.g., Kent et al. (2018b): Seoul, South Korea, and Swindon, United Kingdom, da Silveira et al. (2022): São Paulo, Brazil], and then combine morphometric estimates of $z_0$ and $z_d$ with surface-layer similarity theory, evaluate the resulting flux-profile relationships against flux-tower observations (section 3). Section 4 discusses these findings and how they compare to those from previous research, and Section 5 concludes with the major findings of this work and discusses implications for future research.

## Methods

### Observations and Quality-Control Procedures

**Fig. 1:**
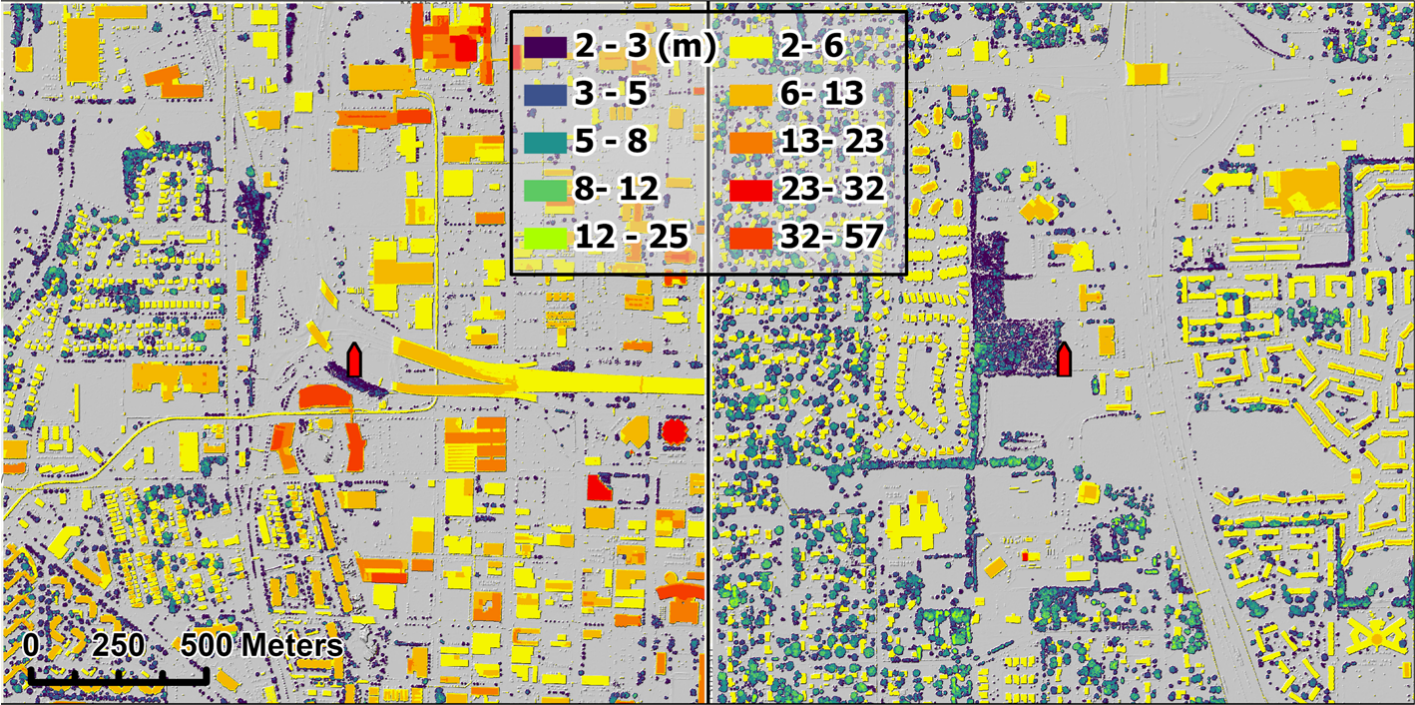
The RESM showing roughness elements $\ge 2$-m for US-INc (a) and US-INg (b). Vegetation is denoted as shades in the left legend column, while built structures are denoted as shades shown in the right column. The LiDAR point cloud data comes from the USGS and the Central Marion County LiDAR survey from 2016

The two sites analyzed in this work are affiliated with the Indianapolis Flux Experiment (INFLUX; Davis et al. 2017) under the Ameriflux identification US-INc and US-INg. These are communication towers with eddy-covariance instruments deployed at 33 m (US-INc) and 41 m (US-INg) above ground level (a.g.l.), respectively, both reporting data at 10-Hz frequency from either a Gill Windmaster (US-INg) or CSAT3 (US-INc) for more information on the INFLUX towers see  , (Horne et al., 2026). The roughness element surface map (RESM; Fig. 1) shows different forms of urbanization around each tower. US-INg is between a highway and a vegetated suburban neighborhood (Fig. 1a). US-INc is surrounded by the Interstate 65-highway network and buildings of various shapes and sizes (Fig. 1b). Because US-INc is sited on an isolated 250-m wide, 10-m high hill, the effective sampling height is 43 m a.g.l..

The RESM shown in Fig. 1 is created by subtracting a digital elevation map (DEM) from a digital surface map (DSM), excluding objects $\le 2$-m. The DEM, DSM, and subsequently RESM are all at 1-m horizontal resolution. The DEM showing ground height only is taken from the 2016 U.S. Geological Survey (USGS 2018). The DSM showing ground plus RE heights is converted from LiDAR point cloud data using the ArcGIS Pro software (ESRI 2024). To guarantee the representativeness of the RESM shown in Fig. 1 and the true RESM during the flux-tower data-collection period, we analyze flux-tower data during the two years closest to the time when LiDAR data were collected (i.e., 2019–2020 at US-INg and 2020–2021 at US-INc). These flux-tower measurements are processed using a combination of the EddyPro software package (LI-COR Biosciences 2024) and in-house Python scripts.

Starting from the 10-Hz data, we utilize quality control methods available within the EddyPro software, including: i) despiking and assessing for dropouts, amplitude resolution, absolute limits, and discontinuities following Vickers and Mahrt (1997), ii) stationarity tests following Foken and Wichura (1996) and Vickers and Mahrt (1997), and iii) comparing integral turbulence characteristics to flux-variance similarity-theory predictions following Foken and Wichura (1996). At the end of these quality-control steps, any 30-minute period with a flag > 5 is removed from subsequent analysis following (Mauder and Foken 2004). When fitting $z_d$ and $z_0$ using anemometric approaches (methods explained in section 2.2) or comparing measurements to similarity theories (methods explained in section 2.5), we further limited data to flag values $\le 3$ (Mauder and Foken 2004). For each 30-minute period, EddyPro outputs turbulence statistics include the mean horizontal wind ($\overline{u}$), friction velocity [$u_{*}=(\overline{u'w'}^2+\overline{v'w'}^2)^{\frac{1}{4}}$, where *u*, $\upsilon$, and *w* represent steamwise, spanwise, and vertical velocity components, respectively], kinematic heat flux ($\overline{w'\theta _v'}$, where $\theta _{\upsilon }$ represents virtual potential temperature), and standard deviation of flow variables ($\sigma _{\phi }$, where $\phi$ can be velocity components or temperature). These statistics are calculated using a block average detrending Foken and Micrometeorology (2008) and also take sonic tilt corrections with angles estimated using a planar fit for each of twelve $30^\circ$ sectors (Rannik et al. 2020). The resulting statistics are used to provide necessary inputs to anemometric and morphometric methods, as well as to calculate the drag coefficient:

1 $$\begin{aligned} C_D = (u_{*}/\overline{u})^2, \end{aligned}$$

and turbulence intensity,

2 $$\begin{aligned} T_\phi =\sigma _\phi /\overline{u}, \end{aligned}$$

where $\phi = u$, $\upsilon$, or *w*. The variation of $C_D$ and $T_{\phi }$ values with wind direction computed for near-neutral conditions can be used to identify flow distortion from nearby buildings and tower frames.

In addition to using EddyPro, we develop in-house Python scripts to estimate the integral time scale ($\tau$) and the degree of anisotropy ($y_b$) for each 30-minute period, which are needed for the investigation of surface-layer similarity theories. Starting from the 10-Hz data, the same quality-control procedures as in the EddyPro software are applied, and the same sector-specific planar-fit rotation matrices are used to perform sonic tilt corrections. The integral time scale is estimated for *u* using two methods: i) identifying the time lag at which the autocorrelation function drops to 1/*e* i.e., the *e*-folding time scale; , (Kaimal and Finnigan, 1994), and ii) integrating the autocorrelation function up to the first zero crossing (Lenschow and Stankov 1986). The degree of anisotropy is defined as $y_b=\sqrt{3}/2(3\lambda _3+1)$ (Banerjee et al. 2007), where $\lambda _3$ is the smallest eigenvalue of the non-dimensional Reynolds stress tensor [$b_{ij}=(\overline{u_i'u_j'}/\overline{u_k'u_k'})-(1/3)\delta _{ij}$, where indices *i* and *j* can be 1, 2, or 3, a repeated index *k* implies a summation over $k = 1$, 2, and 3, and $\delta _{ij}$ is the Kronecker delta]. Here, $y_b$ comes from the barycentric mapping created by Banerjee et al. (2007), a remapping of the original Lumley triangle (Lumley and Newman 1977), which, in itself, is a realizable mapping of the two tensor invariants of $b_{ij}$. The value of $y_b$ ranges from 0 to $\sqrt{3}/2$ with those values closer to $\sqrt{3}/2$ being more isotropic. It should be noted that while the eigenvalues of $b_{ij}$ can be used to visualize various states of the tensor (e.g., Gucci et al. 2025), the values themselves should not be used to interpret the spatial structure of the turbulent flow. Unlike previous estimates of $y_b$ from flux-tower data (e.g., Stiperski and Calaf 2023; Mosso et al. 2024), where a reduced averaging time was used for stable conditions, we use a 30-minute average for all stability conditions. The use of a relatively long averaging time is expected to yield qualitatively similar results as reported , (Mosso et al., 2024).

### Anemometric Approaches of Estimating $z_d$ and $z_0$

The anemometric approaches evaluated here include the temperature variance method (TVM) proposed by Rotach (1994) and the wind variance method (WVM) proposed by Toda and Sugita (2003). Both approaches use the Monin–Obukhov similarity functions for unstable conditions (Tillman 1972; Panofsky et al. 1977; De Bruin et al. 1993; Kustas et al. 1994; Hsieh et al. 1996; Toda and Sugita 2003; Choi et al. 2004):

3 $$\begin{aligned} \phi _T=\frac{\sigma _T}{T_*} = -C_1 \left( C_2-\frac{z'}{L}\right) ^{-\frac{1}{3}}, \end{aligned}$$

4 $$\begin{aligned} \phi _w=\frac{\sigma _w}{u_*} = C_3 \left( 1-C_4 \left[ \frac{z'}{L}\right] \right) ^{\frac{1}{3}}, \end{aligned}$$

where $\sigma _{T}$ and $\sigma _{w}$ are the standard deviations of temperature and vertical velocity, respectively, $z^{\prime }=z_m-z_d$ where $z_m$ is the measurement height a.g.l., $T_{*}=\overline{w'\theta _v'}/u_{*}$ is the characteristic temperature scale for surface-layer heat flux, and $L=-u_{*}^3\overline{\theta _v}/(\kappa g\overline{w'\theta '_v})$ is the Obukohv length (where $\kappa =0.4$ is the von Kármán constant^1^, $g = 9.8$ m $\text {s}^{-2}$ is the gravitational acceleration, and $\overline{\theta _v}$ is the mean virtual potential temperature). This formulation is for the completely unstable regime, given that it captures both the 1/3 scaling law during free convective conditions as well as the convergence of the non-dimensional variance toward a constant as $z'/L$ approaches zero (Tillman 1972). Table 1 summarizes parameter ($C_i$ where $i = 1$, 2, 3, or 4) values fitted using observational data over reported flat homogeneous terrain. Other studies in urban areas have previously fit parameter ($C_i$) values [e.g., Quan and Hu (2009), Beijing, China, Wood et al. (2010); London, U.K., Fortuniak et al. (2013): Łódź, Poland, Falabino and Castelli (2017) Turin, Italy, and Basel, Switzerland, da Silveira et al. (2022): São Paulo, Brazil], however, these urban studies implicitly (i.e., fit without the use of aerodynamic parameters) or explicitly (i.e., fit with a constant set of aerodynamic parameters) assume a value of $z_d$ a priori, which makes interpreting anemometric estimates of $z_d$ using these fitted parameters at a new site a challenge. This fact motivates the choice to use parameters fit to reported flat homogeneous terrain, as was also done in Kent et al. (2018a), when solving for the required $z_d$.

**Table 1:** Constants ($C_1$-$C_4$) used in the temperature variance (TVM) and wind variance (WVM) for estimating $z_d$. All studies reported flat, homogeneous terrain around the tower. Tillman (1972) derived constant values using $\kappa$ = 0.35 compared to 0.4 or 0.41 used by other studies.

Reference	Method	Constant Pair
Tillman (1972)	TVM	$C_1$=0.95 $C_2$=0.050
De Bruin et al. (1993)	TVM	$C_1$=0.95 $C_2$=0.035
Kustas et al. (1994)	TVM	$C_1$=1.10 $C_2$=0.085
Kaimal and Finnigan (1994)	TVM	$C_1$=0.94 $C_2$=0.110
Toda and Sugita (2003)	TVM	$C_1$=0.99 $C_2$=0.060
Choi et al. (2004)	TVM	$C_1$=1.14 $C_2$=0.030
Panofsky et al. (1977)	WVM	$C_3$=1.30 $C_4$=3.00
De Bruin et al. (1993)	WVM	$C_3$=1.25 $C_4$=3.00
Choi et al. (2004)	WVM	$C_3$=1.12 $C_4$=2.80

We use the constant pairs in Table 1 to estimate the displacement height using the tower-based measurements. We estimate the value of $z_d$ for each constant pair by minimizing the root-mean-square error (RMSE) between the observations and similarity functions (3) or (4) during unstable conditions (loosely defined as $z^{\prime }/L$ $\le$ -0.05). We minimize the RMSE by increasing the value of $z_d$ from zero to the measurement height ($z_m$) at an increment of 0.1 m. We select the value of $z_d$ which minimizes the RMSE between observed $T_{*} / \sigma _{T}$ or $\sigma _{w} / u_{*}$ and those predicted by the similarity functions. This incremental method has been used frequently (Rotach 1994; Toda and Sugita 2003; Kent et al. 2017a, 2018b) because a prescribed value of $z_d$ is needed to determine whether an observation period is within the unstable stability criteria. While incrementally increasing the value of $z_d$, we do not consider the $z_d$ estimate for a given wind direction if fewer than fifteen (n$\le$15) measurements meeting the unstable criteria are available using that $z_d$ value. These estimates of $z_d$ are calculated for $10^\circ$ wind sectors. We use the inverse (1/$\phi _T$) of the similarity function to solve for $z_d$, when using the TVM to avoid extreme values of RMSE that distort the optimization (Appendix 1).

We next compute the roughness length for each wind sector. For each $10^\circ$ wind sector, we fit $\overline{u} / u_{*}$ during near-neutral $(\mid z'/L\mid \le 0.05)$ periods using the estimated $z_d$ values to the logarithmic wind profile to obtain $z_0$. Time intervals with wind speeds $\le$ 1 m $\text {s}^{-1}$ are excluded to ensure sufficient well-developed mechanical turbulence (Liu et al. 2009; Kent et al. 2017a, 2018b).

### Morphometric Approaches to Estimating $z_d$ and $z_0$

**Fig. 2:**
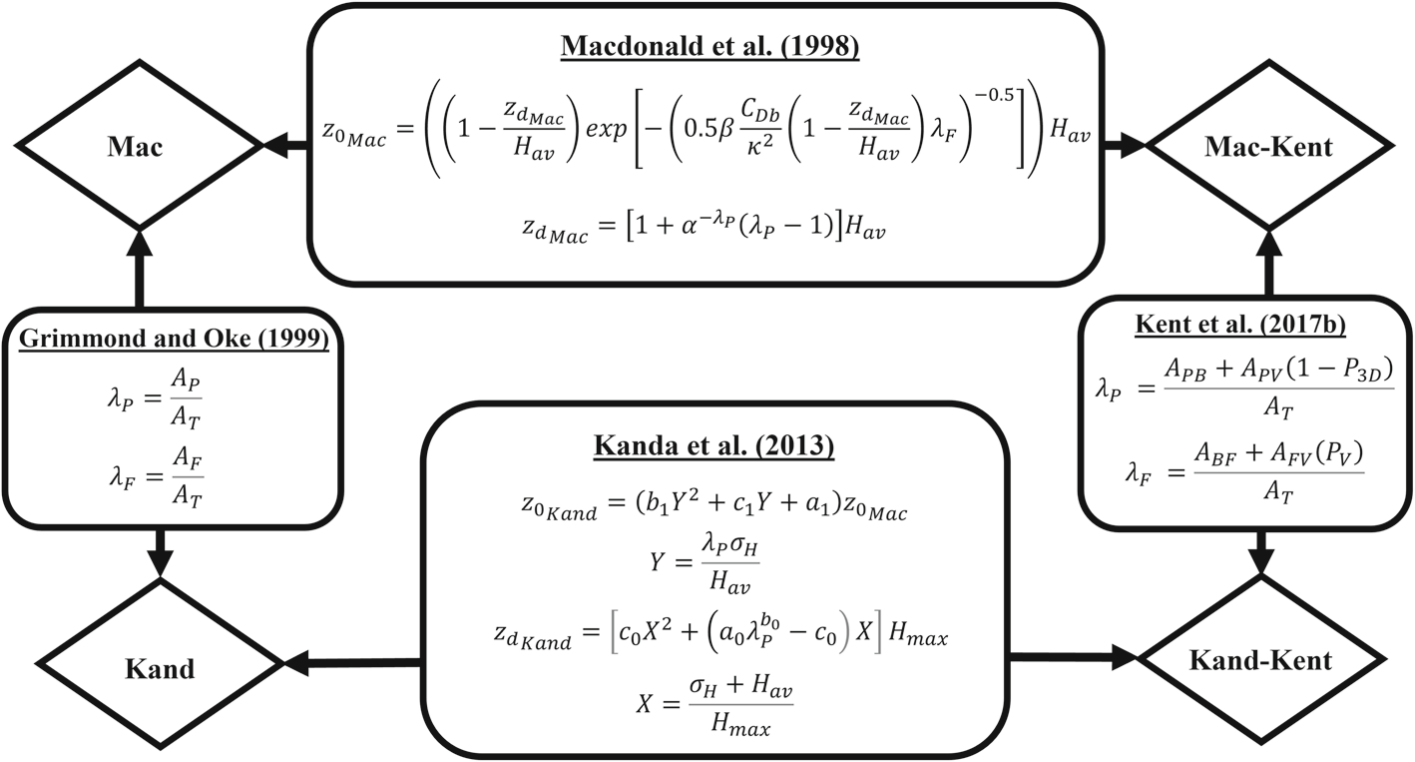
Equations for the morphometric methods presented by Macdonald et al. (1998) and Kanda et al. (2013) used in the morphometric analysis. When calculating $z_0$ via the Kanda et al. (2013) formulation, both the $z_d$ and $z_0$ from Macdonald et al. (1998) are used. Methods of Kent et al. (2017b) separately describe vegetation from buildings. $H_{\textrm{max}}$ is the maximum building height, $C_{Db}$=1.2 is the drag coefficient for buildings, $0 \le X \le 1$, $0 \le Y$, and $\alpha$, $\beta$, $a_0$, $b_0$, $c_0$, $a_1$, $b_1$, $c_1$ are constants of values 4.43, 1, 1.29, 0.36, -0.17, 0.71, 20.21, and -0.77, respectively. $A_{PB}$ and $A_{PV}$ are the planar areas of buildings and vegetation in the tower footprint, $A_{FB}$ and $A_{FV}$ are the frontal areas of buildings and vegetation in the tower footprint, and $A_P$ and $A_F$ are the sum of vegetation and buildings. $P_{3D}$ is the aerodynamic porosity of the vegetation, and $P_V$ is the ratio of drag coefficients of vegetation and buildings ($P_V=\left( -1.251P_{\textrm{3D}}^2+0.489P_{\textrm{3D}}+0.803 \right) / C_{\textrm{Db}}$)

Figure 2 summarizes a total of four morphometric methods to be assessed in this work. The method proposed by Macdonald et al. (1998) (hereafter referred to as *Mac*) and the method proposed by Kanda et al. (2013) (hereafter referred to as *Kand*) are both commonly used morphometric methods. Both methods account for statistics of building morphologies like average height ($H_\textrm{av}$), the planar area index ($\lambda _{\textrm{P}}$), and the exposed frontal area index ($\lambda _{\textrm{F}}$), but *Kand* additionally incorporates the standard deviation of height $\sigma _H$ and maximum height $H_\textrm{max}$. Based on LES results obtained for realistic urban geometries in Japanese cities, Kanda et al. (2013) proposed a new predictive equation capable of replicating $z_d$ values that exceeded $H_\textrm{av}$. Physically, the variability of building heights and drag of taller buildings may lead to $z_d$ values greater than $H_\textrm{av}$, and consequently $H_\textrm{max}$ becomes a more appropriate normalizing length scale for $z_d$ than $H_\textrm{av}$. In addition to *Mac* and *Kand*, we also investigate a modified version of each method that accounts for the difference between vegetation and building (following Kent et al. 2017b, hereafter referred to as *Kent–Mac* and *Kent–Kand*, respectively). Specifically, the expressions of $\lambda _{\textrm{P}}$ and $\lambda _{\textrm{F}}$ original of the form shown in , (Grimmond and Oke, 1999) were modified by Kent et al. (2017b) to consider vegetation as porous media (with the aerodynamic porosity of the vegetation, $P_{3D}$, being 0.6 and 0.2 in winter and summer, respectively). Here, morphometric estimates are only obtained during winter (January, February, and December) and summer (June, July, and August) months to avoid periods of foliar transition. Additionally, morphometric estimates are only obtained for near-neutral conditions, under which these methods were developed.

Note that the comparison of these four morphometric methods performed in this work is not identical to Kent et al. (2018b). In this work, vegetative REs are always present, although treated differently when using various morphometric methods (i.e., as porous media for *Kent–Mac* and *Kent–Kand* but as non-porous media for *Mac* and *Kand*). In Kent et al. (2018b), a majority of the comparisons involved vegetative REs that were only present when using *Kent–Mac* and *Kent–Kand*, but were completely absent when using *Mac* and *Kand*.

Using each morphometric method, we calculate $z_d$ and $z_0$ for each 30-minute period in three steps. First, a footprint model (Kljun et al. 2015) is run, modeling footprint areas contributions from $50\%$, $60\%$, $70\%$, and $80\%$ of the scalar fluxes for a given period at the elevation of $z_m$. The FFP model uses flux-tower measurements, ERA5 boundary-layer heights, and initial guesses for $z_0$ and $z_d$. Next, for each of the footprint areas, the enclosed REs are used to calculate a set of morphological indices like $H_{\textrm{av}}$, $\sigma _{H}$, and $\lambda _{P}$, which can be input into the morphometric formulas to calculate $z_d$ and $z_0$. Finally, each morphometric pair of $z_d$ and $z_0$ is used to generate an updated footprint area, which contributes to the associated percentage of scalar fluxes at $z_m$ on the tower, yielding an updated set of morphological indices and, subsequently, an updated pair of $z_d$ and $z_0$ estimates. Theoretically, this process should be repeated until the estimates of $z_d$ and $z_0$ no longer change. However, such iterative processes are computationally expensive, so we only update $z_d$ and $z_0$ estimates twice. It should be noted that, although not shown, after two iterations general convergence was reached, with the largest difference between the initial guess and the first iteration.

The footprint is calculated using the flux footprint prediction (FFP) model proposed by Kljun et al. (2015). Note that this model is designed for scalar fluxes, not momentum fluxes. Given this, the pixel weighting from the FFP is not applied to roughness elements within the flux footprint percent areas. Additionally, the shape of the scalar footprint predicted by FFP certainly misses some complexities present in reality (see Fig. 1 in Järvi et al. 2009). Any footprint area exceeding the RESM shown in Fig. 1 is excluded.

The initial guesses of $z_d$ and $z_0$ needed to start the footprint estimate are obtained by minimizing the sum of squared residuals between wind speed measured and that predicted by the log-law during near-neutral periods $(\mid (z'/L\mid \le 0.05)$. The residual minimization uses the Nelder-Mead algorithm (Nelder and Mead 1965), which simultaneously solves $z_d$ and $z_0$ for each $10^\circ$ wind sector. Bounds for $z_d$ being within 0–$z_m$ and $z_0$ being within 0–5 m are enforced. For directions impacted by flow distortion from nearby buildings and tower frames (presented in Section 3.1), the Nelder-Mead algorithm is not performed because one does not expect the wind to follow the log-law. Instead, the initial guesses of $z_d$ and $z_0$ are interpolated linearly using values calculated for the two nearest wind sectors that are not impacted by flow distortion.

### Built Structures and Vegetation Around the Towers

To prepare for inputs to morphometric methods (described in Section 2.3), we separate vegetation REs from built structures manually. This manual separation is done in ArcGIS Pro via visual inspection, where we create polygon features around vegetation based on satellite images.^2^ As a result, vegetation and built structures are annotated using different color shades in Fig. 1.

The land cover characteristics at US-INg (Fig. 1a) can broadly be split into east and west sectors. Immediately west of the tower is a forest patch (250 m in the west-east direction and 160 m in the south-north direction), whose $H_{\textrm{av}}$ is 7 m. Further west, the area is primarily suburban, with mature vegetation, where the vegetation ($H_{\textrm{av}}$ = 6 m) is taller than most of the houses ($H_{\textrm{av}}$ = 5 m). Immediately east of the tower is a highway, and across the highway is a residential area ($H_{\textrm{av}}$ = 5 m) with minimal vegetation and some commercial buildings.

At US-INc (Fig. 1b), the surrounding area contains buildings of various shapes and sizes, with the area immediately next to the tower dominated by roadways. The buildings nearest to the tower are approximately 250 m away to the eastern (0–$180^\circ$) and about 300 m away to the western half (180–$360^\circ$), except for a large medical building ($H_{\textrm{av}}$ = 32 m) located 100 m southwest of the tower. Further southwest is a suburban area with a mix of vegetation ($H_{\textrm{av}}$ = 4 m) and built structures ($H_{\textrm{av}}$ = 9 m). To the west and northwest of the tower are a few commercial buildings ($H_{\textrm{av}}$ = 10 m) and another suburban area with a mix of vegetation ($H_{\textrm{av}}$ = 5 m) and houses ($H_{\textrm{av}}$ = 4 m). East of the tower is an area or little vegetation, which consists of built structures that vary in height ($H_{\textrm{av}}$ = 10 m, $\sigma _H$ = 9 m and $H_{\textrm{max}}$ = 58 m). Both towers are assumed to be within the initial sublayer for most wind directions, given the measurement height (US-INc: $z_m\approx 5.4H_\textrm{av}$, US-INg $z_m\approx 7H_\textrm{av}$) and the commonly assumed rule of thumb $2H_\textrm{av}\le z_* \le 5H\textrm{av}$.

### The Surface-Layer Similarity Theories to be Evaluated

The first similarity theory of interest is the surface-layer stress-stain-rate relationship based upon the Monin-Obukhov similarity theory (MOST; Monin and Obukhov 1954) [using diabatic stability corrections proposed by Dyer (1974)], adding a roughness-sublayer correction proposed by De Ridder (2010):

5 $$\begin{aligned} \frac{\partial \overline{u}}{\partial z} = \frac{u_{*}}{\kappa z'} \phi _m\left( \frac{z'}{L}\right) \hat{\phi }_m\left( \frac{z'}{z_*'}\right) . \end{aligned}$$

Here, $z_{*}^{\prime } = z_{*} - z_{d}$ (where $z_{*} \approx 3.5 H_{\textrm{av}}$ is the assumed depth of the roughness sublayer), $\phi _m$ is the MOST similarity function for momentum (which is a function of the stability parameter, $z^{\prime } / L$ Kaimal and Finnigan 1994, chap. 1.3.5),

6 $$\begin{aligned} \phi _m = {\left\{ \begin{array}{ll} \left( 1+16|z'/L |\right) ^{-1/4}, & \text {for } -2 \le z'/L \le 0,\\ 1 + 5\, z'/L, & \text {for } 0 \le z'/L \le 1, \end{array}\right. } \end{aligned}$$

and:

7 $$\begin{aligned} \hat{\phi }_m = 1-\exp {\left( -2.59 z^{\prime } / z_{*}^{\prime } \right) }, \end{aligned}$$

is an empirical correction accounting for the presence of the roughness sublayer. Note that $z_{*} / H_{\textrm{av}} \approx 3.5$ is chosen as the average of values reported for urban areas (2–5; Roth 2000). Integrating (5) from $\overline{u} = 0$ at $z^{\prime } = z_0$ to $z^{\prime } = z_m - z_{d}$ (i.e., the measurement heigh) yields:

8 $$\begin{aligned} \overline{u}\left( z = z_m\right) = \frac{u_{*}}{\kappa }\left[ \ln \left( \frac{z'}{z_0}\right) -\varPsi \left( \frac{z'}{L}\right) +\varPsi \left( \frac{z_0}{L}\right) +\hat{\varPsi }\left( \frac{z'}{L},\frac{z'}{z_*'}\right) \right] , \end{aligned}$$

where

9 $$\begin{aligned} \varPsi (\zeta ) = {\left\{ \begin{array}{ll} \ln \!\left[ \left( \dfrac{1+x^2}{2}\right) \left( \dfrac{1+x}{2}\right) ^2\right] - 2\arctan (x) + \dfrac{\pi }{2}, & \text {for } -2 \le \zeta \le 0, \\ -5\,\zeta , & \text {for } 0 \le \zeta \le 1, \end{array}\right. } \end{aligned}$$

10 $$\begin{aligned} \hat{\varPsi }\!\left( \frac{z'}{L}, \frac{z'}{z_*'}\right) = \phi _m\!\left[ \left( 1 + \frac{\nu }{\mu _m\!\left( \dfrac{z'}{z_*'}\right) }\right) \frac{z'}{L} \right] \frac{1}{\varLambda } \ln \!\left( 1 + \frac{\varLambda }{\mu _m\!\left( \dfrac{z'}{z_*'}\right) } \right) e^{-\mu _m\!\left( \dfrac{z'}{z_*'}\right) }, \end{aligned}$$

where $x = (1-16\zeta )^{1/4}$, $\mu _m=2.59$, $\nu =0.5$, and $\varLambda =1.5$. We can evaluate $\overline{u}$ predicted by (8) against those measured on the towers. Inputs to (8) includes $u_{*}$ and *L* from measurements and estimates of $z_{d}$ and $z_{0}$ using morphometric methods (which are wind-direction dependent). Note that the employment of (6) means that evaluation is limited to the stability range $-2 \le z'/L \le 1$. Theoretically, (5) states a neutral stress-strain-rate relationship being modified by a function of stability, meaning that shear production must remain important in the local turbulent kinetic energy (TKE) budget (i.e., at least half of buoyancy production for unstable conditions and always exceeding buoyancy destruction for stable conditions).

In addition to evaluating $\overline{u}$ predicted by (8), we can also predict turbulence integral length and velocity scales ($\ell$ and $\upsilon$) at $z = z'$:

11 $$\begin{aligned} \ell =\frac{\kappa z' \phi _{\epsilon }^{1/2}}{(\phi _m\hat{\phi }_m)^{3/2}}, \end{aligned}$$

12 $$\begin{aligned} v=u_{*} \left( \frac{\phi _{\epsilon }}{\phi _m\hat{\phi }_m} \right) ^{1/2}, \end{aligned}$$

using (6), (7), and:

13 $$\begin{aligned} \phi _\epsilon = {\left\{ \begin{array}{ll} \left( 1 + 0.5 \left\lvert \dfrac{z'}{L} \right\rvert ^{\tfrac{2}{3}}\right) ^{\tfrac{3}{2}}, & \text {for } \dfrac{z'}{L} \le 0, \\ 1 + 5\,\dfrac{z'}{L}, & \text {for } \dfrac{z'}{L} \ge 0, \end{array}\right. } \end{aligned}$$

the MOST similarity function for TKE dissipation (Dyer 1974). Because a roughness sublayer correction for TKE dissipation is not considered here, and therefore we only predict $\ell$ and $\upsilon$ when the roughness sublayer correction is unimportant (i.e., $\hat{\phi }_{m} \ge 0.9$). This restriction excludes only data at US-INc in the wake of the large medical building, when surface-layer similarity theories are not expected to be applicable in any case.

The second similarity theory of interest is a generalized form of MOST proposed by Stiperski and Calaf (2023) and Mosso et al. (2024), which includes the degree of anisotropy ($y_b$; explained in section 2.1) as an additional non-dimensional parameter into surface-layer similarity functions (e.g., $\phi _{m}$ and $\phi _{\epsilon }$), where from Mosso et al. (2024):

14 $$\begin{aligned} \phi _{\textrm{m}}&= {\left\{ \begin{array}{ll} a(y_b) + b(y_b)\,\zeta , & \zeta \ge 0, \\ \displaystyle \frac{a(y_b) + b(y_b)\,|\zeta |^{\,n(y_b)}}{a(y_b) + |\zeta |^{\,n(y_b)}} + c(y_b)\,|\zeta |^{1/3}, & \zeta < 0, \end{array}\right. } \end{aligned}$$

15 $$\begin{aligned} a&= {\left\{ \begin{array}{ll} 0.76 + 1.5\,y_b, & \zeta \ge 0, \\ 0.012, & \zeta< 0 \ \text {and}\ y_b \ge 0.6, \\ 0.24 - 0.38\,y_b, & \zeta< 0 \ \text {and}\ y_b < 0.6, \end{array}\right. } \end{aligned}$$

16 $$\begin{aligned} b&= {\left\{ \begin{array}{ll} 6.3 - 4.3\,y_b, & \zeta \ge 0, \\ 0.061, & \zeta < 0, \end{array}\right. } \end{aligned}$$

17 $$\begin{aligned} c&= {\left\{ \begin{array}{ll} 0.45 - 0.53\,y_b,&\zeta < 0, \end{array}\right. } \end{aligned}$$

18 $$\begin{aligned} n&= {\left\{ \begin{array}{ll} -0.12 + 6.4\,y_b,&\zeta < 0 \end{array}\right. } \end{aligned}$$

and from Stiperski and Calaf (2023):

19 $$\begin{aligned} \phi _{\varepsilon }&= {\left\{ \begin{array}{ll} a \left( 1 - 3 \zeta \right) ^{-1} - b\,\zeta , & \zeta < 0, \\ \left[ \exp \!\left( a + b \ln (\zeta ) + c \ln (\zeta )^2 \right) \right] ^{1/2}, & \zeta \ge 0, \end{array}\right. } \end{aligned}$$

20 $$\begin{aligned} a&= {\left\{ \begin{array}{ll} 0.371 + 1.811\, y_b, & \zeta < 0, \\ 0.915 + 1.477\, y_b, & \zeta \ge 0, \end{array}\right. } \end{aligned}$$

21 $$\begin{aligned} b&= {\left\{ \begin{array}{ll} 0.908 + 0.166\, y_b^{-1} + 0.006\, y_b^{-2}, & \zeta < 0, \\ 0.176 - 4.278 \ln (y_b) - 4.851 \ln (y_b)^2 - 1.334 \ln (y_b)^3, & \zeta \ge 0, \end{array}\right. } \end{aligned}$$

22 $$\begin{aligned} c&= {\left\{ \begin{array}{ll} -0.172 + 3.488\, y_b - 8.538\, y_b^2 + 6.112\, y_b^3,&\zeta \ge 0. \end{array}\right. } \end{aligned}$$

This “anisotropic MOST” has shown promise for use in complex terrain (Mosso et al. 2024). Here we do not integrate $\partial \overline{u} / \partial z$ vertically to predict $\overline{u}$ (like 8), because we only have measurements of $y_{b}$ at a single height. Thus, the evaluation of anisotropic MOST is limited to turbulent integral scales ($\ell$, $\upsilon$, and $\tau$) and eddy viscosity ($K_{m}$) at $z^{\prime } = z_{m} - z_{d}$. Note that the combination of anisotropic MOST and roughness-sublayer correction has not been validated against experimental or canopy-resolving simulations, which provides another reason why this evaluation is only performed when the roughness-sublayer correction is unimportant (i.e., $\hat{\phi }_{m} \ge 0.9$).

The combination of (11) and (12) predicts an integral time scale ($\tau = \ell / \upsilon$) and an eddy viscosity ($K_{m} = \ell \upsilon$). The $\tau$ values modeled by similarity theory can be evaluated against those derived from observations (using approaches described in section 2.1) to determine if $\tau$ is over- or underestimated by the theory. We can also compare the $\overline{u}$ modeled by similarity theory with those observed at the tower to determine whether the similarity theory modeled $K_m$ is under- or overestimated. The under- or overestimate of $K_{m}$ can be determined given the measured $u_{*}$ in used as an input such that $u_{*}^{2} / \left( \partial \overline{u} / \partial z \right)$, and $\partial \overline{u} / \partial z$ comes from the similarity theory prediction (e.g., 5). Depending on whether $\tau$ and $K_{m}$ are over- or underestimated, we can speculate whether $\ell = \left( \tau K_{m} \right) ^{1/2}$ and $\upsilon = \left( K_{m} / \tau \right) ^{1/2}$ are overestimated or underestimated.

## Results

### Regions of Flow Distortion from Buildings and Tower Frames

**Fig. 3:**
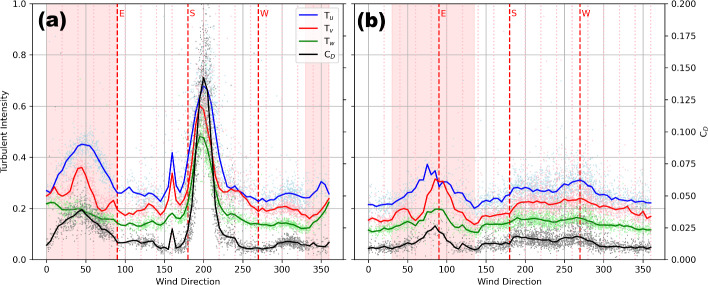
Turbulent intensities and drag coefficients vs. wind direction measured at US-INc (a) and US-INg (b) during neutrally stable regimes $(\mid z/L\mid \le 0.05)$. The solid lines depict $5^\circ$ binned medians. The red shading indicates wind directions where the tower structure impacts flow

Visually, the sharp spikes in turbulence intensity and drag coefficient estimates for near-neutral conditions reveal the impacts of flow distortion by nearby obstacles (Fig. 3). At US-INc, flow distortion from the tower frame is suggested by an apparent rise of these quantities at 330–$360^\circ$ and 0–$90^\circ$, while flow distortion from the nearby medical building is revealed by an even sharper rise of these quantities at 170–$270^\circ$. At US-INg, flow distortion from the tower frame is suggested by an apparent rise of these quantities at 30–$135^\circ$. We also note a mild rise of these quantities at 170–$290^\circ$, which is not caused by flow distortion but the tower being downwind from suburban developments with mature vegetation (see Fig. 1a).

### Anemometric Estimates of $z_d$ and $z_0$

**Fig. 4:**
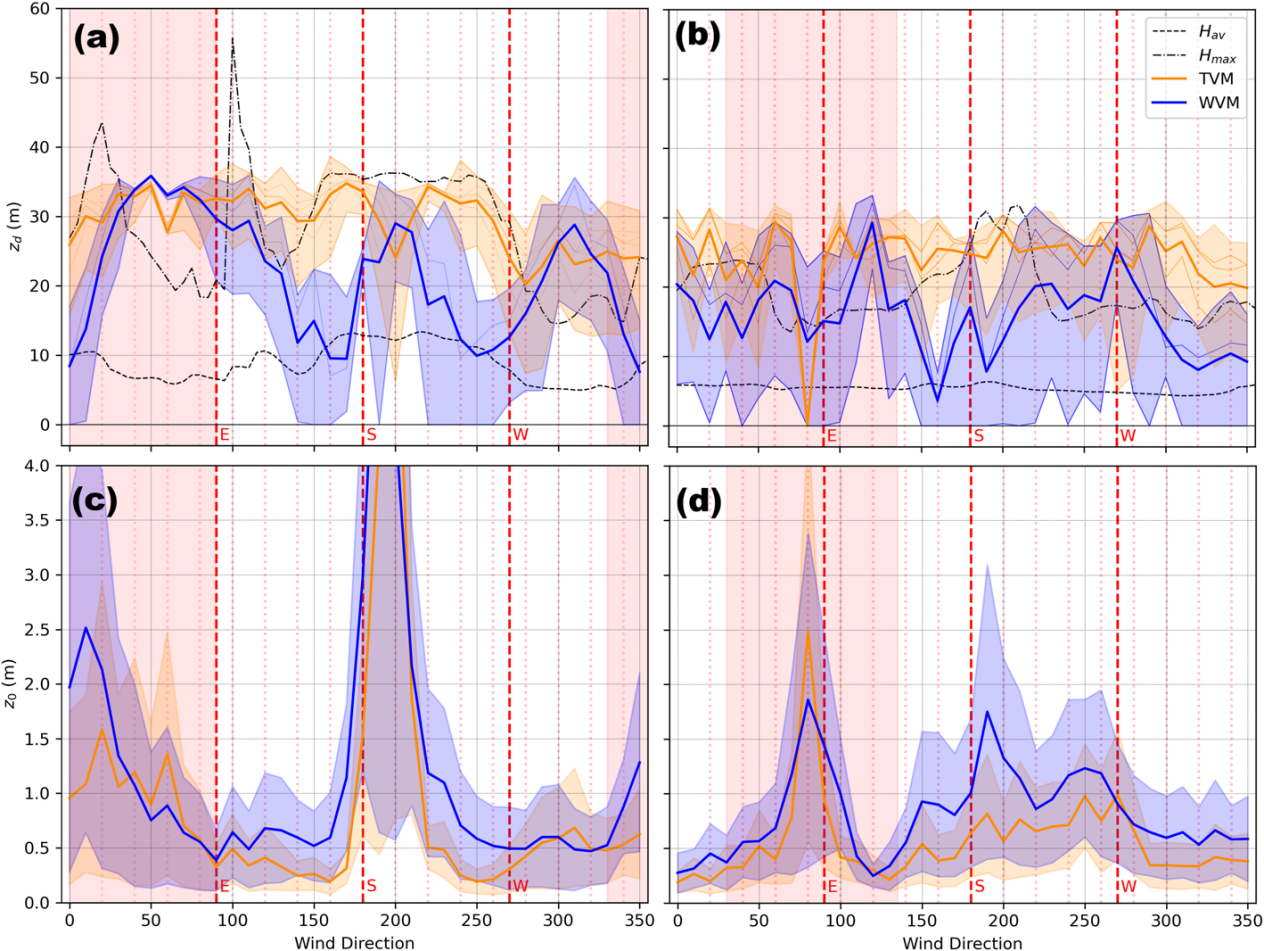
Anemometric estimates of $z_d$ (a, b) and $z_0$ (c, d) for US-INc (a, c) and US-INg (b, d) calculated for each $10^\circ$ sectors. TVM and WVM estimates obtained using various sets of coefficients in Table 1 are represented using thin blue and orange lines, respectively. The mean and range of these TVM and WVM estimates are represented using thick blue lines with blue shaded areas and thick orange lines with orange shaded areas, respectively. In panels (a, b) showing $z_{d}$ estimates, the mean and maximum building heights ($H_{\textrm{av}}$ and $H_{\textrm{max}}$, respectively) are also shown (as black dashed and dash-dotted lines, respectively) for reference purposes. For $z_0$, the shading represents the upper and lower quartiles using the different estimates of $z_d$. The wind directions impacted by flow distortion from tower frames are annotated using light red shaded areas

TVM estimates of $z_{d}$ are higher than WVM estimates of $z_{d}$ on average (Fig. 4a, b). Specifically, TVM estimates of $z_{d}$ obtained using various sets of coefficients in Table 1 range from 6 m to 39 m for US-INc and from 0 m to 32 m for US-INg. If one excludes wind directions impacted by flow distortion from nearby buildings and tower frames, the mean $z_d$ estimates are approximately 28 m for US-INc and 24 m for US-INg. WVM estimates of $z_{d}$ obtained using various sets of coefficients in Table 1 range from zero to 36 m for US-INc and from zero to 33 m for US-INg. If one excludes wind directions impacted by flow distortion from nearby building and tower frames, the mean $z_d$ estimates are approximately 19 m for US-INc and 15 m for US-INg. These results compare well with those reported in recent work, such as Kent et al. (2017a, 2018b) and da Silveira et al. (2022), which report little to no change in the $z_d$ values estimated using the TVM at their urban sites. However, despite their similarities to the reported results of previous research, the estimates of $z_d$ reported here appear unphysical and will be discussed later in Section 4.1.

TVM estimates of $z_{0}$ are lower than WVM estimates of $z_{0}$ on average (Fig. 4c, d). If one excludes wind directions impacted by flow distortion from nearby buildings and tower frames, TVM yields mean $z_0$ estimates of 0.4 m for US-INc and 0.5 m for US-INg, while WVM yields mean $z_0$ estimates of 0.6 m for US-INc and 0.9 m for US-INg. Both TVM and WVM estimates of $z_{0}$ exhibit similar patterns with changing wind direction. These anemometric estimates of $z_0$ are consistent with those reported in previous urban studies, which are on the order of a meter or larger (e.g., Grimmond et al. 1998; Rooney 2001; Giometto et al. 2017; Kent et al. 2017a, 2018b; da Silveira et al. 2022). At US-INc, unrealistically large estimates of $z_0$ (> 3 m) are reported for wind directions impacted by flow distortion from the medical building, which is unsurprising because flows within a building wake are not expected to follow surface-layer scaling. At US-INg, relatively large estimates of $z_0$ are reported for the southwestern sector (170–$290^\circ$), when the tower is downwind from suburban developments with mature vegetation. Given an approximately constant $z_{d}$ (e.g., estimated using TVM), plugging a relatively large $z_{0}$ into the logarithmic wind profile yields a relatively small ratio $\overline{u} / u_{*}$, which implies relatively large turbulence intensity and drag coefficient (consistent with results shown in Fig. 3).

### Morphometric Estimates of $z_d$ and $z_0$

**Fig. 5:**
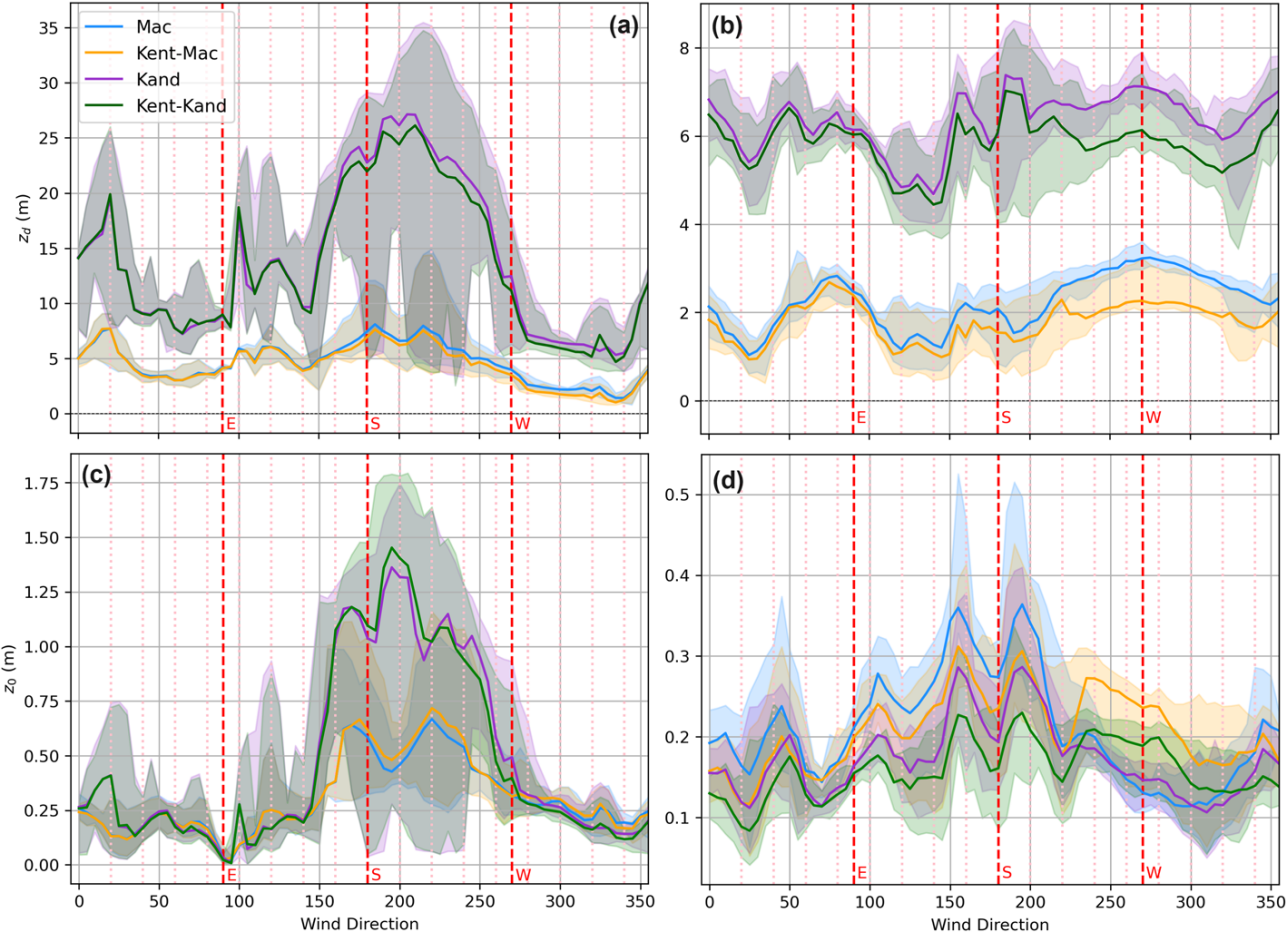
Estimated $z_0$ and $z_d$ from the morphometric methods for US-INc (a, c) and US-INg (b, d) during neutral conditions. The dashed line denotes measurement height ($z_m$), and the dashed black line indicates zero. The darker colored lines denote the median value, and the shading represents the range of values estimated using 50-80% of the footprint areas in binned $5^\circ$ wind sectors

The *Kand* estimates of $z_d$ are roughly 2–3 times those of the *Mac* estimates of $z_{d}$, regardless of whether vegetative REs are treated as porous or non-porous media (Fig. 5a, b, excluding wind directions impacted by flow distortion from nearby buildings and tower frames), consistent with results reported in previous studies (e.g., Kent et al. 2017a, 2018b). These $z_d$ estimates are on average $10\%$–$15\%$ (*Mac*) and $26\%$–$42\%$ (*Kand*) of TVM estimates of $z_d$, and $16\%$–$21\%$ (*Mac*) and $44\%$–$61\%$ (*Kand*) of WVM estimates of $z_{d}$. Overall, $z_{d}$ estimates showed greater variability with wind direction at US-INc compared to those at US-INg.

Compared to $z_{d}$ estimates, $z_0$ estimates show better agreement across all four morphometric methods and minimal directional variability at both sites (excluding wind directions at US-INc affected by flow distortion from the medical building). These $z_0$ estimates are $31\%$–$38\%$ (*Mac*) and $31\%$–$43\%$ (*Kand*) of TVM estimates of $z_0$, and $23\%$–$26\%$ (*Mac*) and $22\%$–$30\%$ (*Kand*) of WVM estimates of $z_0$.

**Fig. 6:**
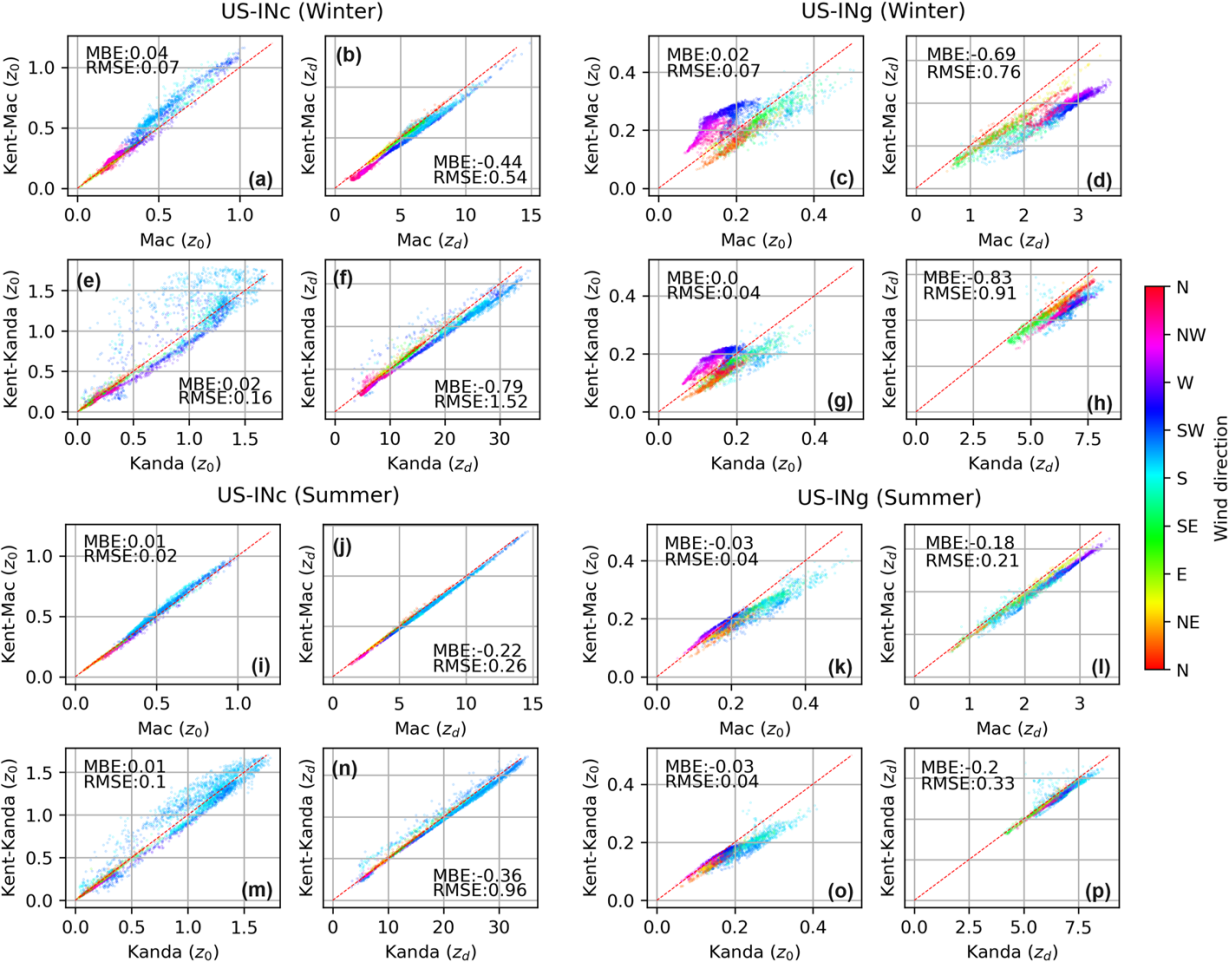
Seasonal comparison between morphometric estimates with and without the methods of Kent et al. (2017b). Values for $z_0$ and $z_d$ estimated for 50–80% footprint fractions are included. The winter season encompasses DJF, and the summer season encompasses JJA. The red dashed line represents a one-to-one line. Values for mean bias error (MBE) and root mean square error (RMSE) are reported in each subplot

Accounting for the porosity of vegetative REs makes negligible impacts on $z_d$ and $z_0$ estimates at US-INc, where nearby vegetation is limited (Fig. 5a, c), but noticeable impacts on $z_d$ and $z_0$ estimates at US-INg when the tower is downwind of vegetated suburban neighborhoods (150–$360^\circ$ in Fig. 5b, d). To further understand the influence of vegetation porosity on morphometric estimates, we separate results obtained for winter and summer months (when $P_{3D} = 0.6$ and 0.2, respectively). According to equations shown in Fig. 2, treating vegetated REs as porous media means increasing $P_{3D}$ from zero to a positive fraction, which decreases $\lambda _\textrm{P}$ and therefore decreases $z_{d}$. For US-INg in winter, $z_d$ estimates obtained using *Kent–Mac* are lower than those obtained using *Mac* (Fig. 6d), while $z_d$ estimates obtained using *Kent–Kand* are lower than those obtained using *Kand* (Fig. 6h), especially when the tower is downwind of the vegetated suburban neighborhood (135–$225^\circ$). The influence of vegetation porosity on $z_d$ is less apparent in summer (Fig. 6l, p) or at US-INc, whose vicinity involves little vegetation (Fig. 6b, f, j, n).

We notice a few instances where accounting for vegetation porosity increases $z_d$ (e.g., Fig. 6f, n, p with southerly winds), which seem inconsistent with equations shown in Fig. 2. This seemingly inconsistent result is caused by different footprint areas used by different morphometric methods, as the second-round footprint estimates are affected by intermediate morphometric estimates of $z_d$ and $z_0$ (see process explained in section 2.3).

The influence of accounting for vegetation porosity on $z_0$ is more complicated than that on $z_d$. According to the equations shown in Fig. 2, $P_V$ peaks at $P_{3D} \approx 0.2$. Consequently, accounting for vegetation porosity has the most significant impact on decreasing $\lambda _\textrm{F}$ in winter. For cases where $z_d$ estimates are insensitive primarily to $P_{3D}$ (e.g., US-INg in summer and US-INc anytime), $z_0$ is expected to depend positively on $\lambda _\textrm{F}$, meaning higher values in summer as compared to the winter, but values that are always lower compared to estimates where Kent et al. (2017b) are not used. Such expectation is sometimes inconsistent with results obtained in this work (e.g., Fig. 5a, e, m). Still, these inconsistencies are predominantly from the wind direction at US-INc impacted by the building wake, and the small footprint area makes the morphometric estimates questionable. For cases where $z_d$ estimates are sensitive to $P_{3D}$ (e.g., US-INg in winter), a decrease in $z_d$ tends to increase $z_0$, whereas a decrease in $\lambda _\textrm{F}$ tends to decrease $z_0$. Such competition leads to non-monotonic dependence of $z_0$ on $P_{3D}$. As a result, we observe an increase in $z_0$ when the tower is downwind of areas with abundant vegetation (225–$315^\circ$ in Fig. 5c, g) but a decrease in $z_0$ in some other directions (0–$180^\circ$ in Fig. 5c, g).

### Surface-Layer Similarity Theory Predictions Employing Morphometric Estimates of $z_d$ and $z_0$

**Fig. 7:**
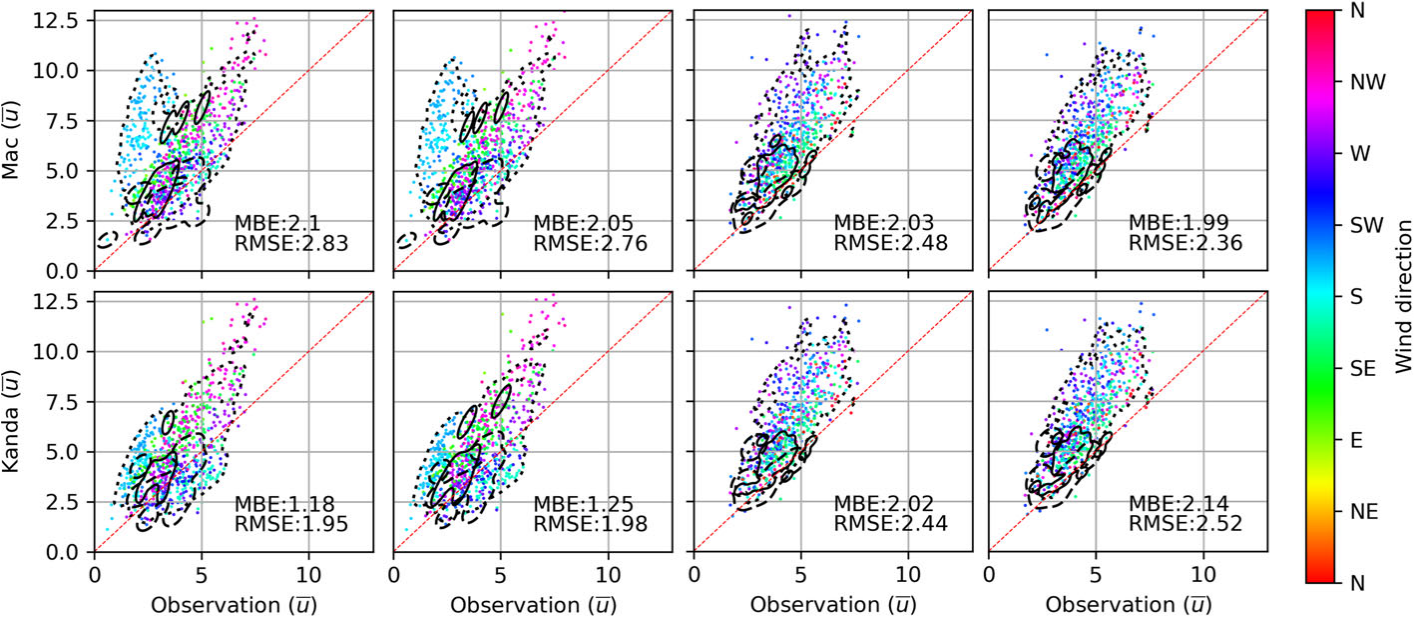
Comparison of thirty-minute mean windspeeds ($\overline{u}$) observations at US-INc (a, b, e, f) and US-INg (c, d, g, h) compared to those estimated using similarity theory and different morphometric methods. Panels b, d, f, and h use the methods of Kent et al. (2017b) to represent vegetation. An outline of the $90\%$ probability density function (PDF) is shown for stability (i.e., $z'/L$) regime groups (dashed: [-2,-0.5], dotted: (-0.5,0.5), solid: [0.5,1]) included to visualize the impact of stability on bias. Values for mean bias error (MBE) and root mean square error (RMSE) are reported in each subplot. Wind directions impacted by the tower frame are removed

Figure 7 shows predictions of $\overline{u}$ at $z = z_m$ using (8) that takes $u_{*}$ from measurements and morphometric estimates of $z_d$ and $z_0$ as inputs. All results, except for better performance given by *Kand* during periods at US-INc impacted by flow distortion from the large medical building (i.e., southerly winds represented by cyan points in Fig. 7a, b, e, f), show clear overall over-predictions of $\overline{u}$, regardless of the choice of morphometric estimates and the associated seasonal and wind-directional variabilities. The over-predictions of $\overline{u}$ are robust, even at US-INg when winds are from the west and measurements are made at $z_m / H_{\textrm{av}} = 8.2$, which should be within the inertial sublayer empirically $z_m / H_{\textrm{av}} \ge 5;$, (Roth, 2000) where similarity theory should be valid.

**Fig. 8:**
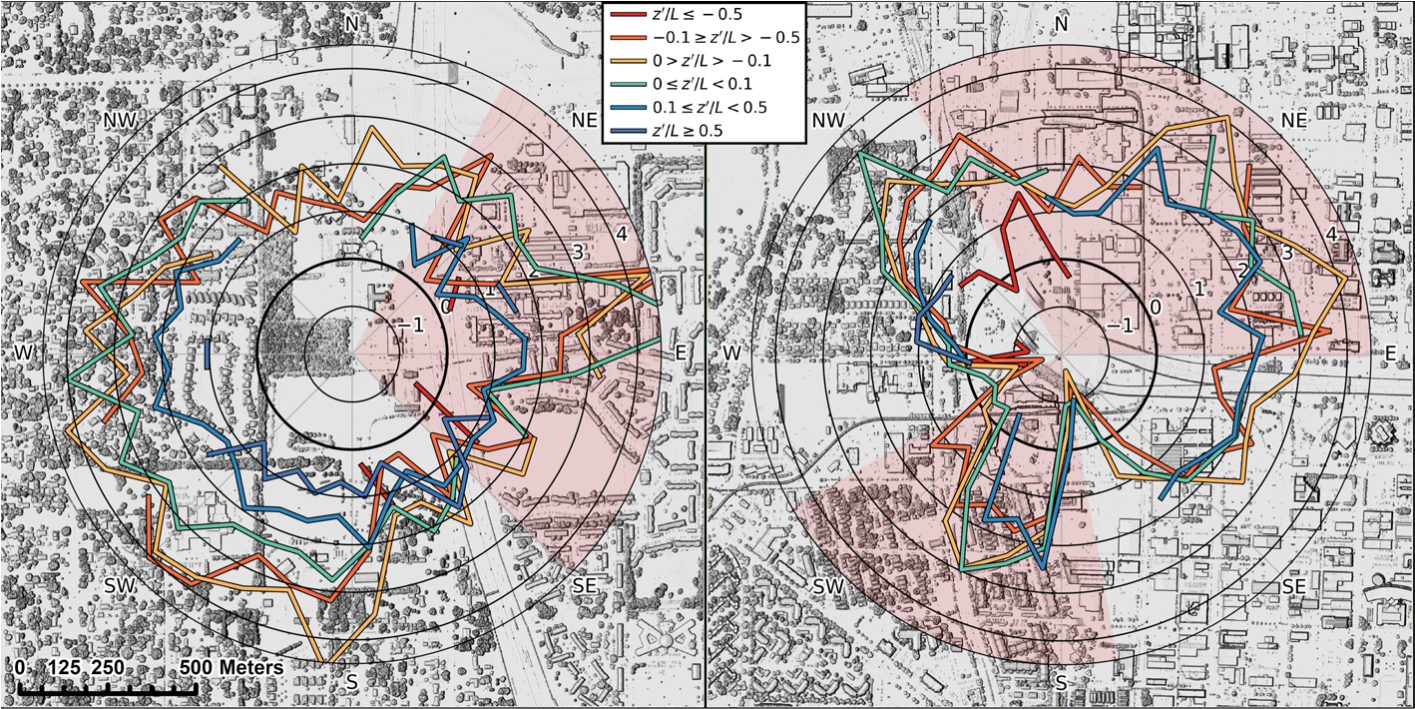
Comparison between $10^\circ$ wind direction binned mean bias error of wind speed for differing stability groups using the *Kent-Kand* aerodynamic parameters at US-INg (left) and US-INc (right). The zero crossing is denoted using a thick black line. Light red shading represents wind directions where flow is distorted by the tower or the large medical building (US-INc). The background is a shaded relief map created from the RE map, including all ground objects

We then investigate the dependence of the $\overline{u}$ over-predictions on stability conditions using results obtained with *Kent–Kand* estimates of $z_d$ and $z_0$ as an example. Figure 8 shows that at both US-INc and US-INg, the most significant over-predictions of $\overline{u}$ occur during periods characterized by $-0.5 \le z^{\prime } / L \le 0.1$ (represented by orange, yellow, and green lines). For a given $u_{*}$, an overestimation of $\overline{u}$ suggests an overestimation of $\partial \overline{u} / \partial z$ and therefore an underestimation of the eddy viscosity, $K_m = u_{*}^{2} / \left( \partial \overline{u} / \partial z \right)$. Thus, the MOST with a roughness-sublayer correction (5) yields overall under-predictions of $K_m$, especially for stability conditions $-0.5 \le z^{\prime } / L \le 0.1$, which are neither too unstable nor too stable. It should be noted that using the stationary tests mentioned in Sect 2.1 we are left with little data in the stability range $-0.5 < z^{\prime } / L$, which is not surprising given weak $\overline{u}$ during these periods which often shifts in direction. Still, during these periods we observe the greatest agreement between similarity predictions and observations (Fig. 7: dashed lines).

**Fig. 9:**
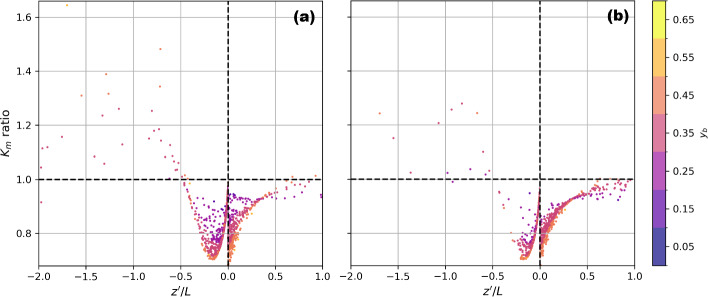
Difference ratio of in eddy viscosity ($K_m$) estimated using the stability functions of Mosso et al. (2024) and Dyer (1974), the former (numerator), the latter (denominator), for each thirty-minute period at a) US-INc and b) US-INg. All points from *Mac*, *Kent-Mac*, *Kand*, and *Kent-Kand* are shown. Points are colored based on the degree of anisotropy ($y_b$), which is calculated from the non-dimensional form of the anisotropic Reynolds stress tensor for each thirty minutes

Compared to (5), the “anisotropic MOST” proposed by Mosso et al. (2024) predicts larger $K_m$ for stability conditions $-2\le z'/L\le -0.5$ but smaller $K_m$ for other stability conditions (Fig 9). For unstable conditions, this increase in $K_m$ through the influence of anisotropy takes into account the role of ABL-scale circulations that are absent in (5), leading to an increase in $K_m$. However, as mentioned, we already see relatively good agreement between (5) and observation under these stability conditions. For other stability conditions, including $-0.5 \le z'/L \le 0.1$ (i.e., the cases with the most significant biases shown in Fig. 8), the decrease in $K_m$ would lead to even larger over-predictions of $\overline{u}$.

**Fig. 10:**
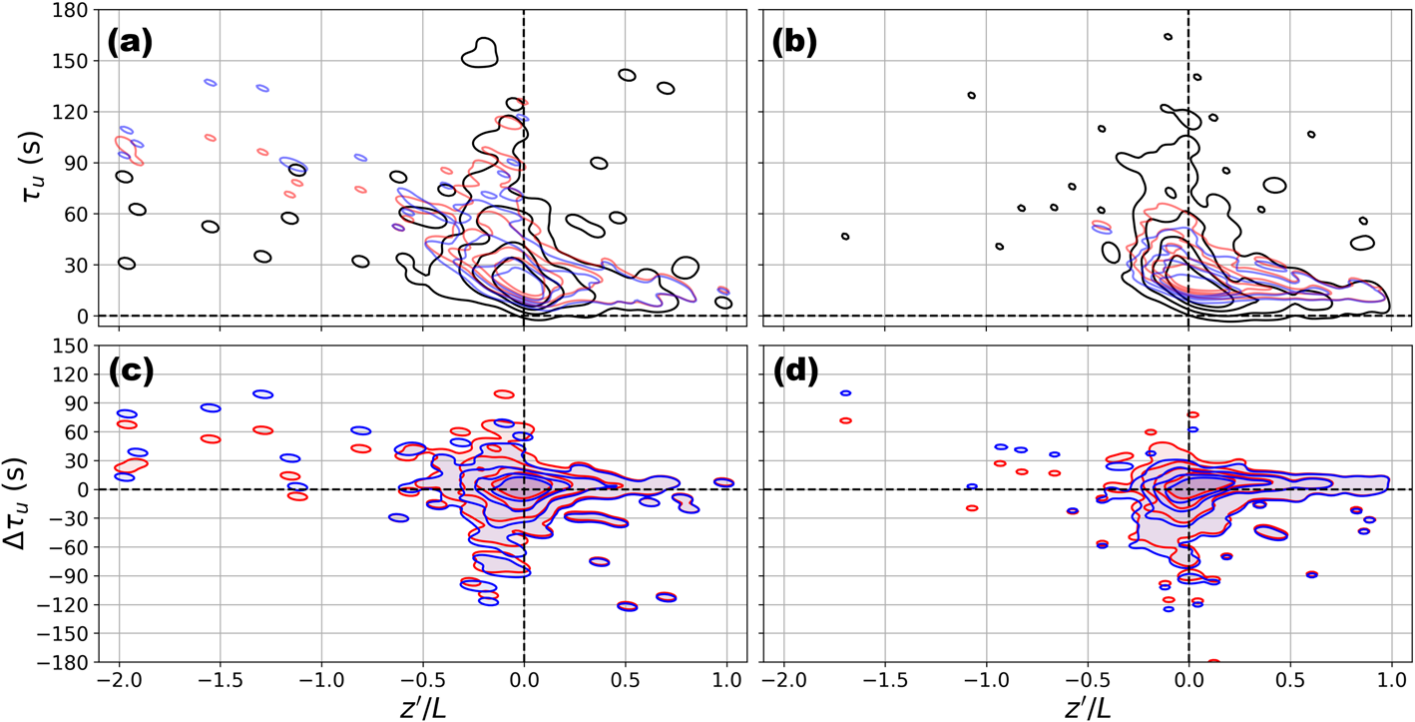
Probability density functions for estimated horizontal integral timescales from measurements (efolding: grey), traditional MOST (red), and MOST from Mosso et al. (2024) (blue) at US-INc (a,c) and US-INg (b,d) using different *Kent-Kand* morphometric estimates. Individual timescale estimates, as determined from measurements, are shown as light gray points in panels a and b. Panels c and d show the differences between the similarity estimate and measurement (measurement subtract similarity estimate). Probability contours in all panels are for $95\%$, $80\%$, $60\%$, $40\%$ of the data

Next, we investigate the predictions of the integral time scale ($\tau$), which can be combined with the predictions of $K_m$ to understand the predictions of integral length and velocity scales, $\ell$ and $\upsilon$. Estimates of $\tau$ in the field are insensitive to the choice between the *e*-folding time and the autocorrelation function integrated to the first zero crossing (methods explained in section 2.1). Surface-layer similarity theory predictions of $\tau = \ell / \upsilon$ are insensitive to the choice between (5) and the “anisotropic MOST” (comparing red and blue contours in Fig. 10); nor are they sensitive to the choice of morphometric estimates of $z_d$ and $z_0$ (calculation explained in section 2.5).

Similarity theory estimates of $\tau$ match well with observations at both towers for stability conditions $0.5\le z'/L \le 1$ (Fig. 10). During these stable periods, the magnitudes of $\overline{u}$ are often overestimated, but the biases are overall smaller than those during near-neutral periods (comparing solid curves to dotted in Fig 7). The combination of underestimated $K_m$ and well-predicted $\tau$ suggests an underestimated $\ell = \left( K_m \tau \right) ^{1/2}$ and underestimated $\upsilon = \left( K_m / \tau \right) ^{1/2}$.

For stability conditions $-2\le z'/L \le -0.5$, similarity theories over predict $\tau$ at both towers (Fig. 10). The combination of a $K_m$ matching well with observations and an overestimated $\tau$ suggests an underestimated $\upsilon$. Compared to (5), the “anisotropic MOST” predicts a greater $K_m$ during these unstable periods, but also a slightly higher $\tau$ making it hard to deduce if the newer formulation will help reduce the underestimation of $\upsilon$.

For stability conditions $-0.5 \le z^{\prime } / L \le 0.5$, similarity theory estimates of $\tau$ deviate from observations in both positive and negative directions (Fig. 10). The largest discrepancies (i.e., 120 s and above) are observed when $\tau$ values are underestimated. The combination of underestimated $K_m$ and underestimated $\tau$ suggests underestimated $\ell$.

## Discussion

### Potentially Unrealistic Anemometric Estimates of $z_d$

Both TVM and WVM estimates of $z_d$ frequently exceed $H_\textrm{max}$ (Fig. 4a, b), which makes little physical sense if $z_d$ is related to momentum exchange on the surface of REs (Thom 1971; Jackson 1981; Zilitinkevich et al. 2008). Such unrealistically large $z_d$ estimates in urban settings have been questioned by previous studies (e.g., Grimmond et al. 1998; Christen 2005; Kent et al. 2017a). For TVM, Grimmond et al. (1998) hypothesized that the large seemingly non-physical estimates of $z_{d}$ could be attributed to a lack of thermal homogeneity within the upwind fetch violating the assumptions of the TVM (Rotach 1994). Using these two methods to estimate $z_d$ implicitly assumes the surface layer is horizontally homogeneous, which is likely violated to some degree in urban landscapes.

**Fig. 11:**
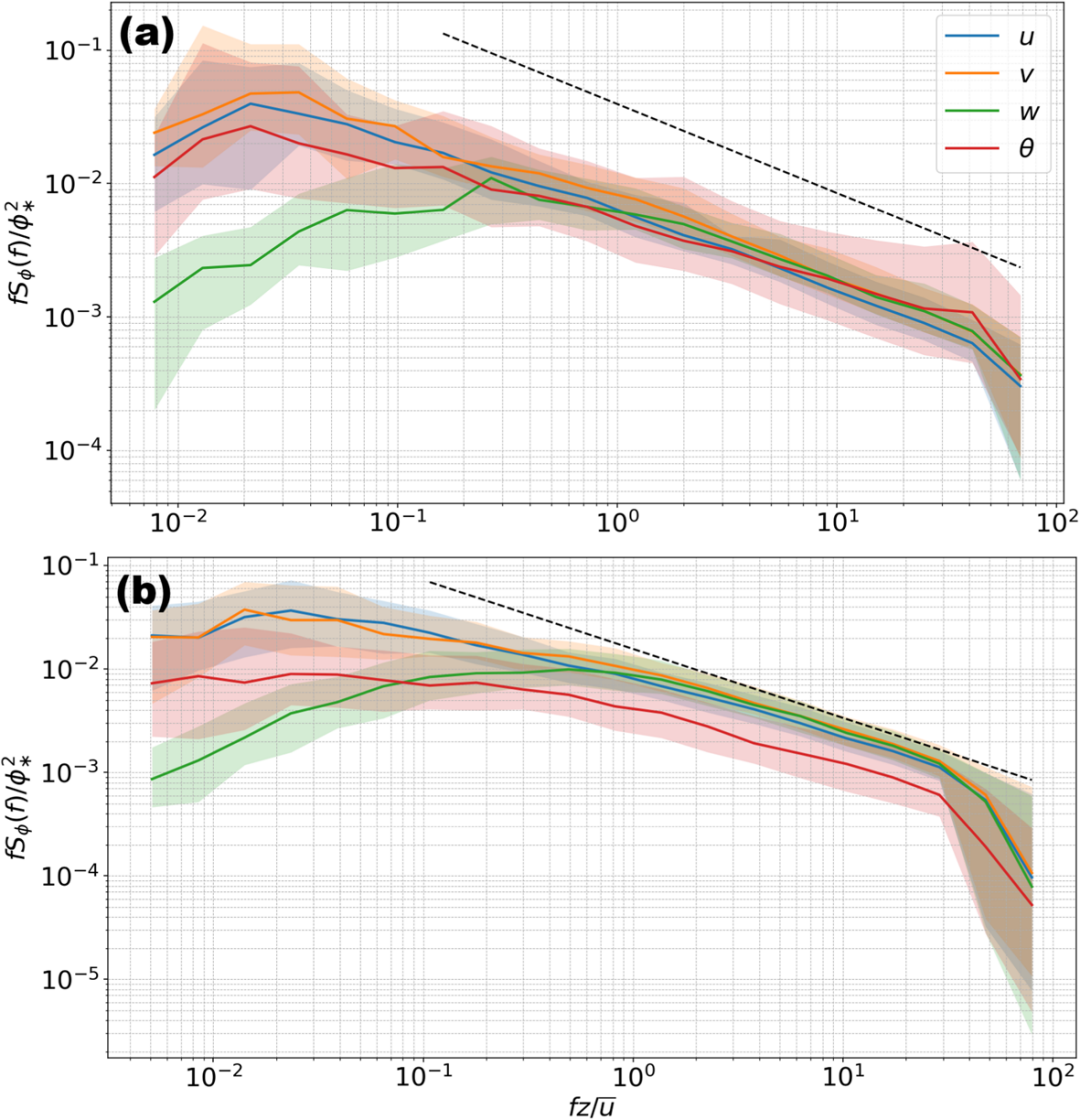
Binned and normalized one-dimensional power spectra for *u*, $\upsilon$, *w*, and $\theta$ from July 2021 for wind directions between $250- 300^\circ$ US-INg (a) and US-INc (b) for $z/L\le -0.1$. The $\phi$ is a stand-in variable for spectra variables, and $\phi _*$ is the associated surface layer scaling variable (i.e., $u_*$ or $T_*$). Colored lines indicate median values for frequency bins, and the envelope around the line corresponds to the 25th and 75th quartile range. The -2/3 slope (dashed line) associated with the inertial subrange is also shown

Whether momentum and scalars share the same $z_{d}$ remains debated (Hicks et al. 1979; Garratt 1992), and the answer could be stability dependent (Raupach et al. 1979; Zilitinkevich et al. 2008). Our results showing TVM estimates higher than WVM estimates on average suggest that they likely do not share the same $z_{d}$ for convective conditions. Grimmond et al. (1998) showed similar results and hypothesized this could be due to the discrepancy between the average source and sinks of momentum and temperature. Another concern is raised by the conflict between the magnitude of anemometric estimates of $z_{d}$ and the characteristic scales of temperature and velocity variances (Fig. 11). Specifically, TVM estimates of $z_{d}$ are higher than WVM estimates on average, implying that temperature variance is scaled with a smaller $z^{\prime } = z_{m} - z_{d}$ than vertical velocity variance. This implication contradicts to the expectation that vertical velocity fluctuation is induced by instability development in vertical direction, which is limited by the distance from the underlying surface ($z^{\prime }$), whereas temperature fluctuation is induced by instability development in all three directions, where horizontal motions are scaled by ABL depth ($z_{i} \gg z^{\prime }$). The expectation that temperature variance is characterized by a larger length scale than vertical velocity variance is consistent with temperature spectrum peaking at a lower frequency than the vertical velocity spectrum (Fig 11). Here, a relatively low spectrum peak frequency implies a relatively large characteristic time scale and, therefore, a relatively large characteristic length scale.

For WVM, we are not aware of any obvious violations of theoretical assumptions that are responsible for the unrealistically large estimates of $z_d$. Nevertheless, vertical velocity often involves the largest uncertainties among variables measured using a sonic anemometer. A primary source of uncertainties is the sonic tilt estimate (Vickers and Mahrt 2006). In this work, we apply planar fit to twelve $30^\circ$ sectors separately, because the recommended practice of using $\ge 90^\circ$ sectors (Wilczak et al. 2001) is not suitable for the heterogeneous urban setting of interest. The mean-wind estimates, which are taken by planar fit as inputs, also involve uncertainties because: i) wind becomes relatively weak and "meanders" (i.e., shifts in direction frequently) for convective conditions, ii) the two stationarity tests employed by EddyPro are imperfect (see detailed explanation in Pan and Patton 2017), and iii) the transducer shadow correction performed within EddyPro is incomplete (as the algorithm is available for only Gill WindMaster at US-INg but not for Campbell Scientific CSAT3 at US-INc).

Given the potentially unrealistic estimates of $z_{d}$, the subsequent estimates of $z_0$ are also questionable. More information on the unrealistic estimates of $z_0$ using anemometric methods is discussed below. Thus, the anemometric estimates of $z_d$ and $z_0$ obtained in this work (and likely for other urban sites) should be interpreted with caution. Note that our results do not support the recommendation of using TVM (in preference to other anemometric or even morphometric methods) in urban settings made by da Silveira et al. (2022).

These unrealistic anemometric estimates of $z_d$ and potentially $z_0$ could explain the often large differences when compared to morphometric estimates, which have also been shown in previous studies. For example, our results at US-INg and US-INc agree with those from Seoul Forest Park, shown in Kent et al. (2018b), where morphometric estimates are consistently smaller than anemometric estimates. The Swindon site, in Kent et al. (2018b), exhibits a similar underestimate when comparing morphometric estimates to anemometric estimates of $z_0$, albeit to a lesser degree than observed at US-INg and US-INc.

### Potentially Questionable Representation of Vegetation Impacts on Morphometric Estimates of $z_0$

**Fig. 12:**
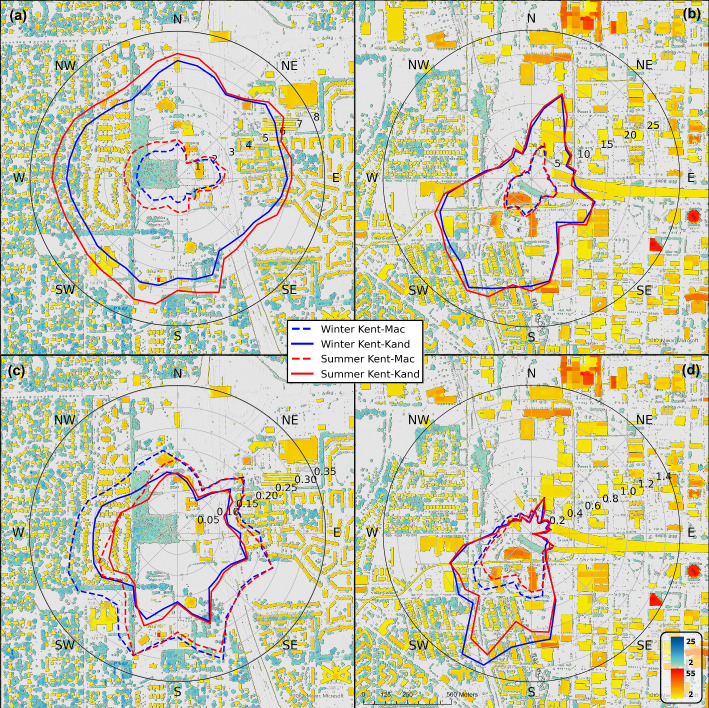
Difference in seasonal morphometric estimates of $z_d$ (a, b) and $z_0$ (c, d) incorporating the methods from Kent et al. (2017b) where $P_\textrm{3D}$ is assumed 0.2 in summer and 0.6 in winter. Lines are $10^\circ$ binned $z_0$, and $z_d$ means for US-INg (a, c) and US-INc (b, d). Cooler background colors (blue/green) indicate vegetation heights, and warmer colors (orange/red) indicate built structure heights

Figure 6 shows that at US-INg (where vegetation impacts are noticeable), morphometric methods that account for vegetation porosity (i.e., *Kent–Mac* and *Kent–Kand*) report smaller $z_d$ values but larger $z_0$ values overall during winter months (i.e., defoliated) than during summer months (i.e., foliated). Such seasonal differences are better visualized in Fig. 12, where $z_d$ estimates during winter months are smaller than those during summer months regardless of the wind direction (comparing blue and red lines in Fig. 12a), and $z_0$ estimates during winter months are larger than those during summer months particularly for winds from 250–$360^\circ$ (comparing blue and red lines in Fig. 12c). These seasonal differences are consistent with those reported by Kent et al. (2018b) for two eddy-covariance sites, one in an urban park in Seoul, South Korea, and the other in a suburban residential neighborhood in Swindon, United Kingdom.

The seasonal differences in morphometric estimates of $z_0$ induced by the changes in vegetation porosity seem inconsistent with LES results of a vegetated neighborhood in Vancouver, Canada (Giometto et al. 2017, who reported smaller $z_0$ values in winter than in summer). Kent et al. (2018b) attributed these inconsistencies to a difference in canopy structure and a non-monotonic dependence of $z_0$ on $\lambda _F$. Whereas in a sparsely packed canopy, $z_0$ increases with decreasing porosity, and in a densely packed canopy, it decreases. According to expressions shown in Fig. 2, $z_{0Mac}$ increases with increasing $\lambda _F$ (i.e., more total frontal area), but decreases with increasing $z_{dMac}$ (i.e., less of the total frontal area inducing drag), which increases with $\lambda _P$. As mentioned in Section 2.3, $P_V$ (i.e., the ratio of vegetative drag coefficient and building drag coefficient) peaks at $P_{3D} \approx 0.2$ using the form proposed by Kent et al. (2018b), meaning that $\lambda _F$ also peaks at $P_{3D} \approx 0.2$. On the other hand, $\lambda _P$ decreases monotonically with increasing $P_{3D}$, implying that the indirect dependence of $\lambda _P$ and therefore $z_{dMac}$ on $\lambda _F$ is non-monotonic. This non-monotonic dependence of $z_{dMac}$ on $\lambda _F$ leads to the non-monotonic dependence of $z_{0Mac}$ on $\lambda _F$. Subsequently, in the expression of $z_{0Kand}$, both $z_{0Mac}$ and *Y* (which is a function of $\lambda _P$) depend non-monotonically on $\lambda _F$, leading to a non-monotonic dependence of $z_{0Kand}$ with $\lambda _F$.

Such reasoning, however, may not fully explain the inconsistency in seasonal changes of $z_0$ comparing the morphometric estimates at US-INg and LES results obtained for Vancouver, Canada. This is because, for example, the canopy characteristics of the upwind neighborhood at US-INg (Appendix 2) are more similar to the canopy characteristics of the LES (Giometto et al. 2017, Table 2) than the canopies investigated by Kent et al. (2018b). Thus, at least the current morphometric formulations of $z_0$ remain questionable for applications to urban sites that are noticeably impacted by vegetation.

### The Overestimation of $\overline{u} / u_{*}$ by Surface-Layer Similarity Theories

The overestimation of $\overline{u}/u_*$ in urban environments by surface-layer similarity theories is not unique to the results shown in Section 3.4. Kent et al. (2018b) reports a positive bias in winds estimated using the logarithmic wind profile compared to flux observations at their site in Swindon, England (Kent et al. 2018b, see Fig. 8). Their study showed that representing vegetation using the methods of (Kent et al. 2017b) helps but does not entirely correct for the positive bias between observed and estimated wind speed during neutral conditions. Similarly, numerical modeling studies (e.g., Talbot et al. 2012; Reames and Stensrud 2017; Sarmiento et al. 2017) have also reported a positive bias in the wind speeds or a negative bias in $u_*$ in urban areas when using the single-layer urban canopy model (SLURM; Kusaka et al. 2001) in the Weather Research and Forecasting (WRF; Skamarock et al. n.d.), which uses the Macdonald et al. (1998) equations for $z_d$ and $z_0$ in the parameterization. A study by Theeuwes et al. (2019) uses the Macdonald et al. (1998) equation with and without stability and roughness sublayer corrections on $z_0$ to compare observations in the roughness sublayer to similarity formulations. They also show an underestimation of $\overline{u}/u_*$ by MOST with and without the De Ridder (2010) roughness sublayer correction during neutral to unstable conditions (defined as $-4\le H_\textrm{av}/L\le 0.1$) at a tower in Gothenburg (top measurement at $z_m=1.8H_{\textrm{av}}$), Sweden which aligns with the overestimate shown here at US-INg and US-INc. At a second tower in Basel (top measurement at $z_m=1.5H_{\textrm{av}}$), Switzerland, they report a mix of overestimates and underestimates that varied with wind direction and stability. It should be noted that integrating the form of stability dependence, as was done by Theeuwes et al. (2019), would act to decrease $z_0$ for $z'/L\le 0$, which would act to increase the bias observed at US-INg and US-INc.

### Linking the Underestimation of $\ell$ by Surface-Layer Similarity Theories and the Unrealistically Large Anemometric Estimates of $z_d$

Results presented in Section 3.4 show an frequent underestimation of $K_m$ particularly for the stability regime $-0.5 \le z'/L \le 0.5$. Combining the underestimated $K_m$ with the underestimated $\tau$ (e.g., the largest discrepancies observed for near-neutral conditions) suggests the underestimated $\ell$. Combining the underestimated $K_m$ with well-predicted $\tau$ (e.g., for stable conditions) suggests underestimated $\ell$ and underestimated $\upsilon$. Combining the underestimated $K_m$ with overestimated $\tau$ (e.g., for some unstable and near-neutral conditions) suggests underestimated $\upsilon$. If motions under these unstable and near-neutral conditions are solely turbulent, then an underestimation of $\upsilon$ is likely associated with an underestimation of $\ell$. In summary, results presented in Section 3.4 suggest an overall underestimation of $\ell$ by surface-layer similarity theories. Such underestimation of $\ell \propto z^{\prime } = z_m - z_d$ is consistent with the unrealistically large anemometric estimates of $z_d$ discussed in Section 4.1.

### Recommended Estimates of $z_d$ and $z_0$ for Using Existing Surface-Layer Similarity Theories in Urban Environments

Surface-layer similarity theories, such as (8), are necessary to parameterize diagnostic variables at the surface (e.g., $U_\textrm{10}$) and surface-atmosphere exchange (e.g., $u_*$) in numerical weather or climate simulations. Before correcting the underestimated $\ell$ and $\upsilon$, which may require multiple decades of effort in urban flux-tower observations and LES work, we can enhance the performance of existing similarity theories by recommending estimates for $z_d$ and $z_0$ and potentially data sets which could be integrated into the numerical models. Morphometric estimates have already been integrated into existing numerical weather prediction models. For example, the single-layer urban canopy model in WRF, which currently uses the *Mac* method. While none of the existing theories are perfect, the morphometric method *Kent-Kand* appears to be both geophysically reasonable and incorporates seasonal variability. Furthermore, with the increasing availability of remote sensing data, it is becoming easier to obtain a high-resolution description of the urban surface, which can be combined with a morphometric method like *Kent-Kand* and integrated into numerical weather models such as WRF.

As discussed in Section 4.1, the anemometric estimates of $z_d$ are unrealistically large, making the subsequently anemometric estimates of $z_0$ potentially questionable. Thus, using morphometric estimates of $z_d$ to fit $z_0$ to observations could yield more geophysically feasible estimates. Here we take morphometric estimates of $z_d$ and then explore what $z_0$ values lead to the best performance of (8). Specifically, for each observational period, we input into (8) the observed $\overline{u} / u_{*}$ and the median $z_d$ estimate of the associated $5^\circ$ bin, and then solved for $z_0$ using the Nelder–Mead algorithm.

**Fig. 13:**
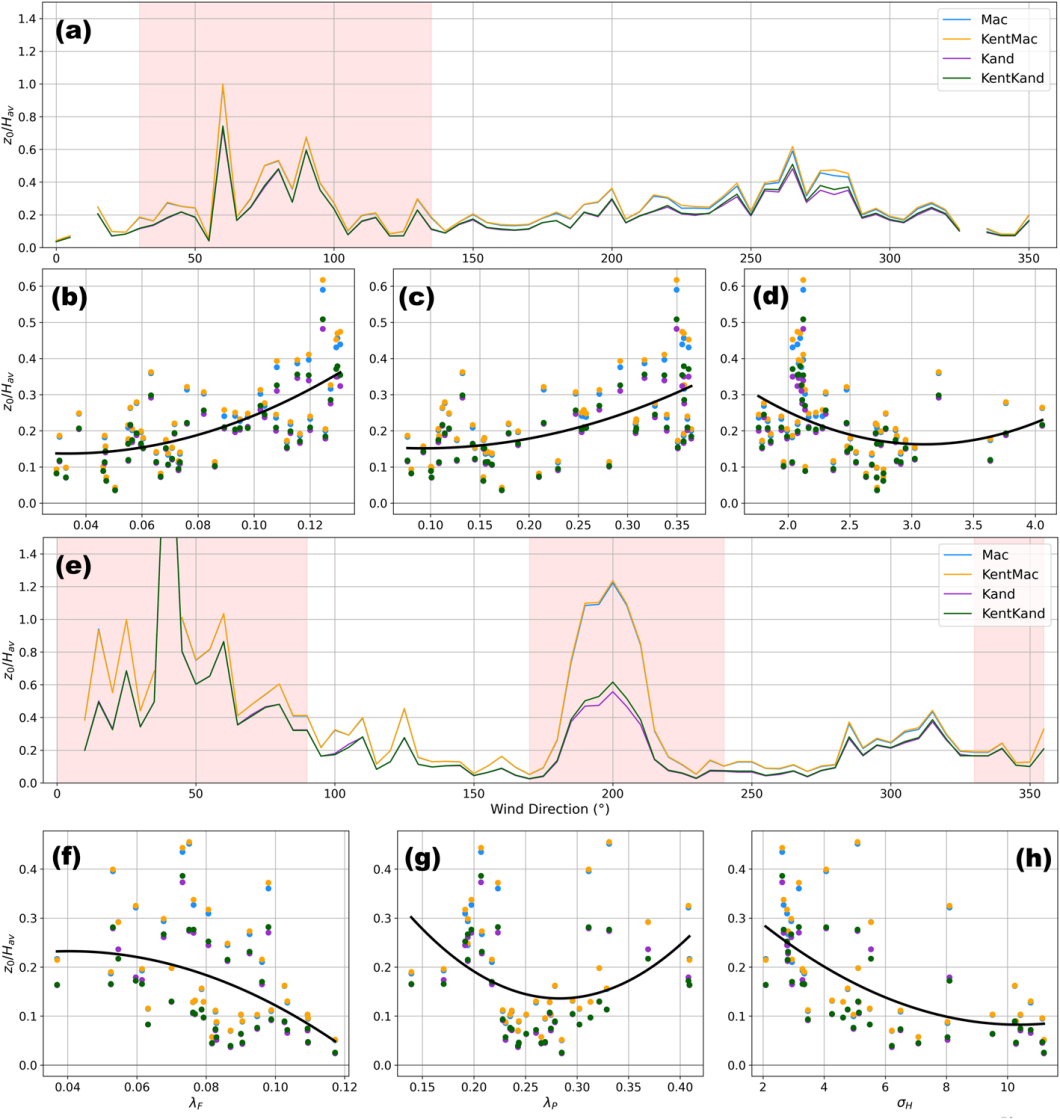
Comparison of median $z_0$ values normalized by $H_{av}$ fit using morphometric estimates of $z_d$ against wind direction, $\lambda _P$, $\lambda _F$, and $\sigma _H$. The values of $H_{av}$, $\lambda _P$, $\lambda _F$, and $\sigma _H$ come from $5^\circ$ binned mean values as shown in Fig. 17. Panels a, b, c, and d show results from US-INg, and panels e, f, g, and h show results from US-INc. A second degree polynomial is fit through the data shown in panel b, c, d, f, g, and h. The red shading in panels a and e shows wind directions impacted by either the tower frame or building (US-INc). These impacted wind directions are not included in the comparison against morphological statistics shown in panels b, c, d, f, g, and h

Figure 13 shows the results of median $z_0$ obtained for each $5^\circ$ bin, which are sometimes large and appear unrealistic (e.g., those reaching $0.4 H_\textrm{av}$ at US-INg for wind directions between $255^\circ$ and $285^\circ$). For reference, given the values of $\lambda _\textrm{F}$ that never exceed 0.15 and $\lambda _\textrm{P}$ that never exceed 0.5 (Fig. 13b, c, f, g), neither morphometric expressions nor previous observations have reported such large $z_0$ values [see Fig 4.24 in Oke et al. (2017) and Fig 6 in Grimmond et al. (1998)]. Moreover, the relationships between $z_0$ and other morphological parameters do not always agree with those implied by expressions shown in Fig. 2. For example, morphometric methods expect $z_0$ to increase with increasing $\lambda _\textrm{F}$ at relatively low $\lambda _\textrm{F}$ values, and to decrease with increasing $\lambda _\textrm{F}$ at relatively high $\lambda _\textrm{F}$ values, as discussed in Section 4.2. Such expectation is not seen at US-INc, where $z_0$ decreases monotonically with increasing $\lambda _\textrm{F}$, regardless of the choice of morphometric estimates of $z_d$ (Fig. 13f). For another example, the *Kand* method expects $z_0$ to increase with increasing *Y*, a positive definite linear function of $\sigma _\textrm{H}$. Such expectation is not discernible at US-INg (Fig. 13d), and is conflicted by $z_0$ that decreases with increasing $\sigma _\textrm{H}$ at US-INc (Fig. 13h).

## Conclusion

Here, we compare the aerodynamic parameters (i.e., $z_0$ and $z_d$) estimated using multiple anemometric and morphometric methods at two urban flux-tower sites in Indianapolis, IN. Results show that the differences in $z_0$ and $z_d$ are likely related through the limitations of surface-layer similarity theories. Specifically, using morphometric estimates of $z_0$ and $z_d$ (which are geophysically reasonable), the similarity theory overestimates $\overline{u}/u_*$ (also reported by Kent et al. 2018b). The overestimation is likely related to an underestimate of $\ell$ and *v* even at elevations theoretically (based on the rule-of-thumb $2H_\textrm{av} \le z_*\le 5H_\textrm{av}$) in the inertial sublayer.

The large discrepancies between observations and similarity theory predictions during near-neutral periods are particularly puzzling. Potential causes of such large discrepancies include: i) nonstationarity that has not been detected using the algorithms embedded in EddyPro, and ii) horizontal heterogeneity in the urban environments. Previous studies of neutral flows have demonstrated that the roughness heterogeneity can generate secondary circulations (e.g., Anderson et al. 2015) and non-negligible dispersive motions within and above the urban canopy (Giometto et al. 2016; Blunn et al. 2022; Li and Bou-Zeid 2019; Akinlabi et al. 2022). These secondary circulations and dispersive motions have been shown to impact measurements of $u_*$ over less complex landscapes (Eder et al. 2015a, b). A recent study by Akinlabi et al. (2022) modeling a downtown region of Boston, MA, USA, shows that these motions can induce horizontal variability at heights above $H_\textrm{max}$ and up to $30 H_\textrm{av}$, which may help explain why our observations at heights up to $8 H_\textrm{av}$ deviate from similarity theory predictions significantly.

The efforts of Kent et al. (2017b) on distinguishing the aerodynamic impacts of vegetation from bluff body objects represent a valuable first step in advancing morphometric methods for mixed urban canopies, although the potential benefits of such efforts are overshadowed by the consistent overestimates of $\overline{u}/u_*$. The seasonal change in $z_0$ predicted by the morphometric analysis and the LES simulation of Giometto et al. (2017) do not entirely agree; however, both the LES simulation and morphometric methods have some level of uncertainty and assumptions, making it difficult to determine which of the two captures the correct seasonal change in $z_0$. Continued refinement of the morphometric representation could benefit from additional comparisons to canopy-resolving numerical simulations of mixed urban canopies, similar to those presented by Giometto et al. (2017). This is particularly important, given that there has been a recent increase in interest in expanding green infrastructure (e.g., planting trees, converting abandoned lots into parks) in cities, which means the abundance of mixed urban canopies will likely increase in the coming decades.

## Figures and Tables

**Fig. 14:**
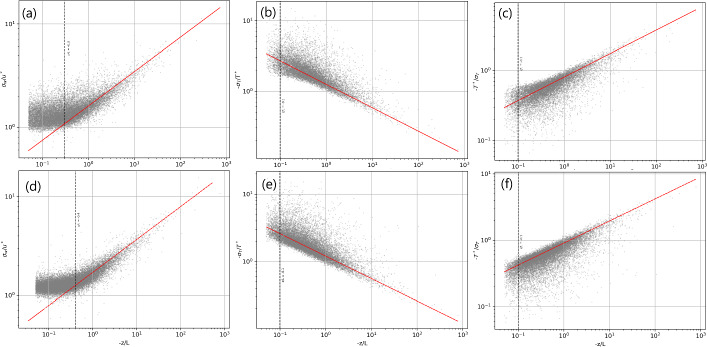
Observations at US-INc (a, b, c) and US-INg (d, e, f) of the standard deviations of temperature or vertical velocity scaled by the surface layer velocity (i.e., $u_{*}$) or temperature. Panels c and f show the inverse of the typical non-dimensional group. The dashed red line has a slope of $\pm 1/3$, showing where observations follow the surface layer similarity theory. Axes are logarithmically scaled, and a dashed black line is included to illustrate where observations begin to follow the $\pm 1/3$ law

**Fig. 15:**
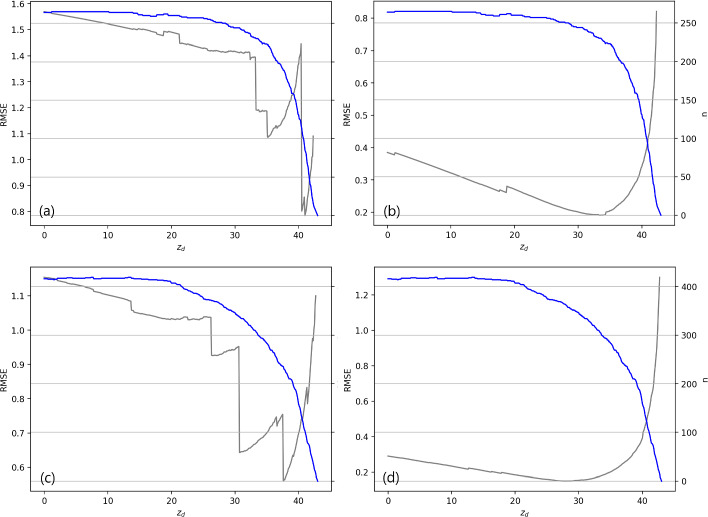
Examples at US-INc (a, b) and US-INg (c, d) when estimating $z_d$ using the $-\sigma _T/T_*$ (a, c) vs. the inverse $-T_*/\sigma _T$ (b, d). The grey line is the root mean squared error (RMSE) (left y-axis) and the blue line is the number of observations (right y-axis) under unstable conditions ($z'/L \le$ -0.05)

**Fig. 16:**
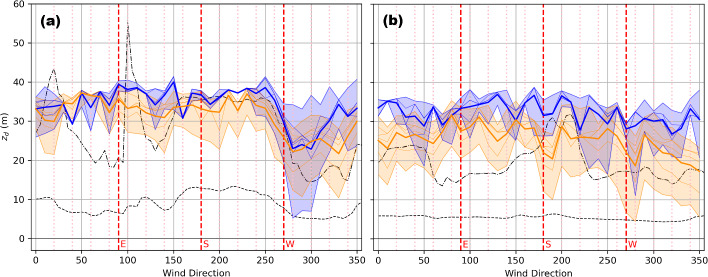
Estimated $z_d$ from the temperature variance method (TVM) using $\phi _T$ ($-\sigma _T/T_*$: blue) and ${\phi _T}^{-1}$ ($-T_*/\sigma _T$: orange) for US-INc (a) and US-INg (b). The median $5^\circ$ bins RE statistics $H_{\textrm{av}}$ (dotted) and $H_{\textrm{max}}$ (dashed) from Fig. 17 are shown. The dark blue (orange) line is the mean value for $10^\circ$ wind sectors. Thin blue (orange) lines represent estimates from different constant pairs. For $z_d$, the shading represents the range of values using differing constant pairs for each $10^\circ$ wind sector

**Fig. 17:**
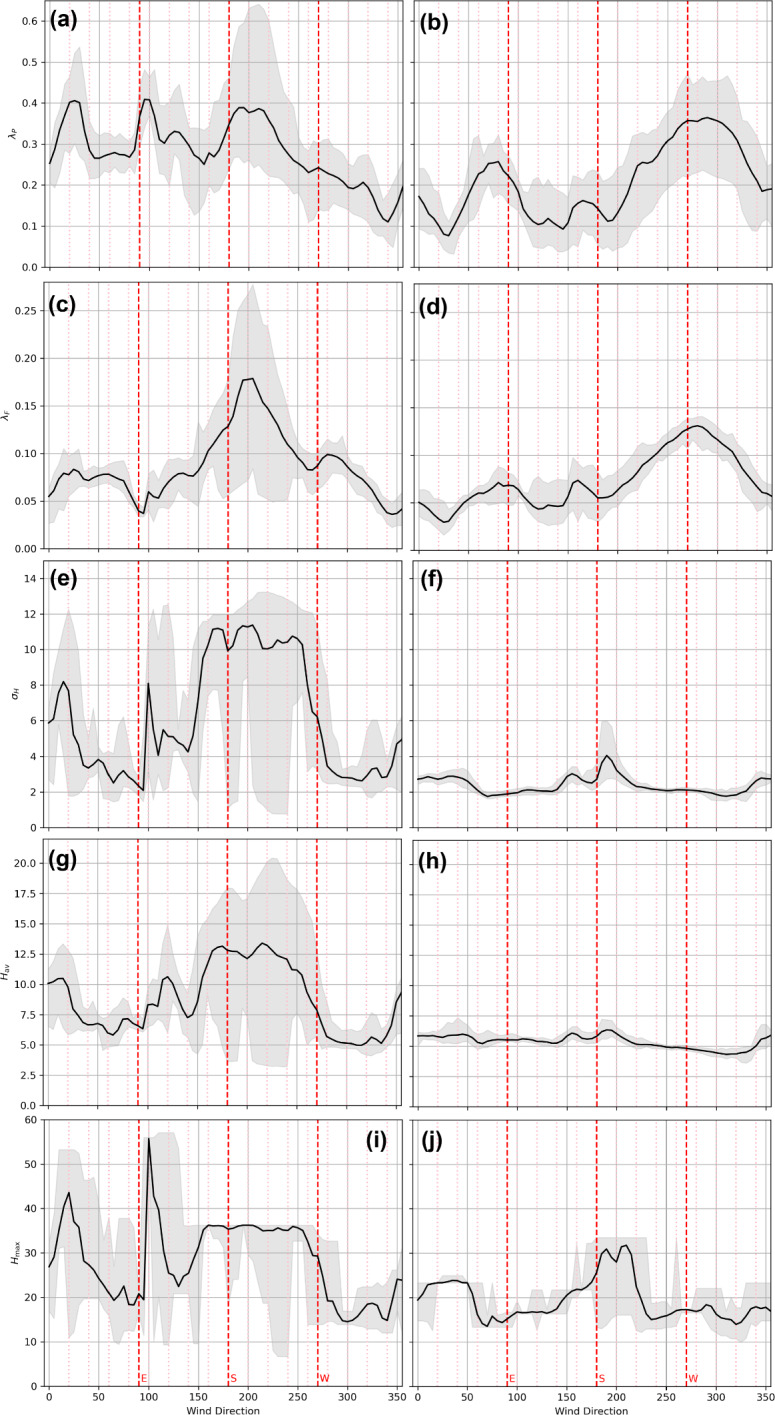
morphological statistics of the upwind REs with in footprint source area at US-INc (a, c, e, g, i) and US-INg (b, d, f, h, j). These values are estimated using the $50\%-80\%$ footprint source area from the Kljun et al. (2015) FFP model. The black line represents the $5^\circ$ binned mean values and gray shading represents the range for each of the same $5^\circ$ bins

## Data Availability

Data from the two flux towers analyzed in this study are available through Penn State’s Datacommons (10Hz: https://doi.org/10.26208/2NT2-RS82, 30min: https://doi.org/10.26208/E8CE-ZH47). All other data from publicly available data sources used in the study can be found through the associated citation. For any other inquiries regarding the manuscript, please reach out to any of the listed authors.

## Appendix 1: The reason to use $\phi _T^{-1}$ rather than $\phi _T$ when solving for $z_d$ using TVM methods

For TVM methods described in Section 2.2, using the classic non-dimensional group $\phi _T$ instead of ${\phi _T}^{-1}$ will change the estimated $z_d$. Figure 14 shows that observations at both sites follow the scaling laws of surface layer similarity theory in unstable and free convective conditions. If $\phi _T$ is used when minimizing the RMSE, abrupt discontinuities appear in the RMSE minimization curve (Fig. 15: grey curve), consistently selecting $z_d$ values at upper limits near z=$z_m$. This is caused by the nature of the function $\phi _T$ and the methods used here. As we iteratively increase $z_d$, the observations that fall within the $z'/L \le$ -0.05 stability range change to include more unstable observations, fewer in number (Fig. 15: blue curve). These more unstable observations are lower in magnitude; thus, the smaller number of smaller $\phi _T$ magnitudes causes the RMSE to be lower and a preferential selection of $z_d$ values close to $z_m$. Instead, if we use ${\phi _T}^{-1}$, the issues with using $\phi _T$ are avoided (Figs. 15 and 16).

## Appendix 2: Morphological statistics of upwind REs

Here, the morphological statistics of REs upwind of each tower (discussed in Section 4.2) are reported with changing wind directions (Fig. 17). These are estimated using varying percent areas (e.g., $50\%$, $60\%$, $70\%$) of the Kljun et al. (2015) FFP model, where all elements within that percent area are equally weighted.

Wind directions influenced by the large medical building at US-INc exhibit the largest values and highest variability of $\sigma _H$, $H_{\textrm{av}}$, $\lambda _P$, and $\lambda _F$ as the footprint size is changed. This can be attributed to the overall small size of the footprint from this direction due to slower wind speeds and larger estimates of $z_d$. This small footprint size means potentially large impacts from single REs in the footprint, making morphometric approaches problematic (Kent et al. 2017a).

## Footnotes

1 Please note we use a constant value of $\kappa$ for the current methodology but acknowledge previous research which highlights a potential dependence on aspects like stability (e.g., Andreas et al. 2006).

2 We have attempted automated methods, but they were unable to yield the desired distinguishing accuracy.
